# Design, synthesis, and biological evaluation of vanillin–piperidone hybrids with potent anticancer activity and a favourable genotoxic profile

**DOI:** 10.1039/d6ra03816f

**Published:** 2026-07-03

**Authors:** M. Krishna Vamsi, Jhansi Mamilla, Ramya Bandari, A. Niranjan Kumar, Afra Fatima, Nidhi Maurya, Kotesh Kumar J., K. V. N. S. Srinivas, Sunil Misra, Suaib Luqman, Amtul Zehra, B. Balakishan

**Affiliations:** a Phytochemistry Division, CSIR-Central Institute of Medicinal and Aromatic Plants, Research Centre Boduppal Hyderabad-500092 India koteshkumarj@cimap.res.in kvn.satyasrinivas@cimap.res.in +91-40-27202602; b Department of Applied Biology, CSIR-Indian Institute of Chemical Technology Hyderabad-500 007 India; c Bio-Prospection and Product Development, CSIR-Central Institute of Medicinal and Aromatic Plants Lucknow-226 015 India; d NMR Division, CSIR-Central Institute of Medicinal and Aromatic Plants Lucknow-226 015 India; e Academy of Scientific and Innovative Research (AcSIR) Ghaziabad Uttar Pradesh 201002 India

## Abstract

A new series of 1-ethoxycarbonyl-3,5-bis(benzyl/alkyl vanillin)–4-piperidone analogues were synthesized *via* a two-step process involving aldol condensation of vanillin with 1-ethoxycarbonyl-4-piperidone, followed by O-alkylation using diverse aromatic and aliphatic halides. Structural characterization of the resulting compounds (3 and 4a–4m) was confirmed by NMR and HR-MS analyses. Among the synthesized derivatives, compounds 3 and 4k exhibited the most potent and selective cytotoxicity. Compound 3 demonstrated IC_50_ values of 2.97 µM (SKBR3), 4.4 µM (DU145), and 20.5 µM (HEK-293), while compound 4k showed strong activity against HepG2 (2.27 µM) and DU145 (5.01 µM). Genotoxic assessment revealed that 4k maintained a favourable safety profile, with chromosomal aberration and micronucleus frequencies remaining low or only mildly elevated at higher doses, and a moderate, dose-dependent decrease in mitotic index. Mechanistic studies indicated G0/G1 cell cycle arrest in CHO-K1, DU145, and HepG2 cells, along with significant apoptosis induction in HepG2 (Sub-G1: 13.9%). Although its antioxidant potential was moderate relative to rutin, 4k exhibited dose-dependent free radical scavenging. Antidiabetic screening identified compounds 1, 3, 4c, and 4m as effective α-glucosidase inhibitors (52.6–58.5%; IC_50_ = 4.27–4.75 µg mL^−1^), comparable to acarbose. Antimicrobial evaluation showed broad-spectrum activity for compound 1, and compound 4k displayed membrane-stabilizing effects similar to quercetin. In conclusion, these multifunctional vanillin–piperidone hybrids especially compound 4k demonstrate significant anticancer, antidiabetic, and antimicrobial potential, further strengthened by their confirmed non-genotoxic profile.

## Abstract 2

Evaluation of the biological activities of 1-ethoxycarbonyl-3,5-bis(benzyl/alkyl vanillin)-4-piperidone analogues synthesized by aldol condensation followed by O-alkylation.

## Graphical abstract

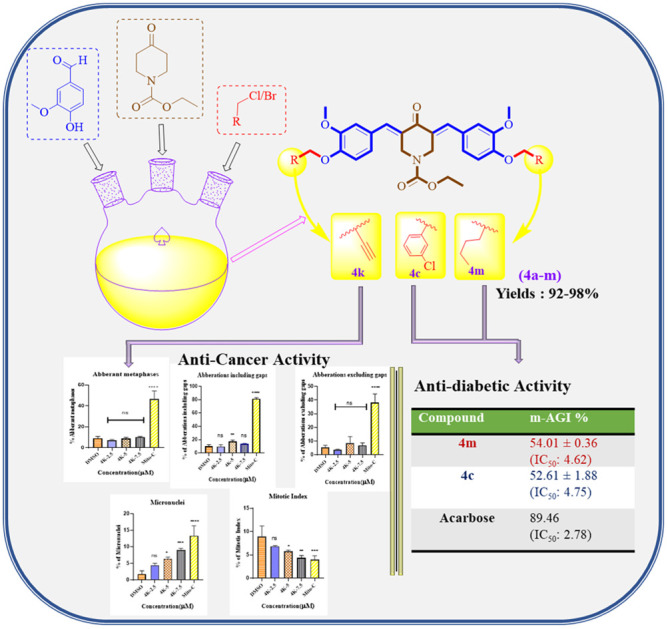

## 1 Introduction

The advancement of hybrid molecules has emerged as a potent strategy in contemporary organic and medicinal chemistry, enabling the design of compounds with enhanced or synergistic biological properties.^1,2^ Among the promising scaffolds used in such hybrid systems, vanillin (4-hydroxy-3-methoxybenzaldehyde) and 4-piperidone stand out for their unique structural features and diverse biological activities.^3,4^ Vanillin is a naturally derived aromatic aldehyde widely utilized as a versatile building block in synthetic chemistry due to its reactive functional groups, including phenolic, aldehydic, and methoxy moieties.^5^ These groups enable various chemical transformations, allowing the synthesis of structurally diverse semi-synthetic derivatives.^6^ Vanillin and its analogues have been extensively studied for their broad spectrum of pharmacological properties, including antimicrobial, antioxidant, and anticancer activities, underscoring their relevance in pharmaceutical and industrial applications.^6–9^ Researchers can strategically design vanillin-based molecules with improved efficacy, reduced toxicity, and targeted therapeutic profiles.^10,11^ Similarly, 4-piperidone, a six-membered heterocyclic compound bearing a ketone group, has attracted considerable interest as a scaffold in medicinal chemistry. Its cyclic amide structure allows for extensive derivatization, giving rise to analogues with notable anticancer, antimicrobial, and analgesic properties.^3,12–15^ Furthermore, its functional groups provide a chemically flexible platform for conjugation with other bioactive fragments, enabling the rational design of novel hybrid molecules.^16,17^ The combination of vanillin and 4-piperidone into a single molecular framework represents a compelling approach in drug discovery and advanced material design. The resulting hybrids contain dual pharmacophores that potentially exhibit improved pharmacokinetics, increased bioactivity, and enhanced selectivity.^18^ Synthetic strategies such as Schiff base formation,^19^ Mannich-type reactions,^20,21^ and Michael addition have been successfully employed to construct such conjugates, offering precise control over the resultant molecular architecture and functionality.

Piperidone-based curcuminoids, particularly 3,5-diarylidene-4-piperidone scaffolds and their *N*-methyl or mono-carbonyl derivatives, have emerged as chemically stable, drug-like mimics of curcumin that preserve and often enhance its broad-spectrum anticancer activities while circumventing curcumin's intrinsic limitations in stability and bioavailability. Recent synthetic and structure–activity relationship studies^3,22^ demonstrate that strategic molecular modifications, including halogenation, aryl substitution, and *N*-alkylation, markedly improve their cytotoxic potency in many human cancer cell lines. The possible mode of action of these piperidone curcuminoids induces mitochondrial-mediated apoptosis, evidenced by caspase activation, PARP cleavage, and loss of mitochondrial membrane potential, while concurrently suppressing key pro-survival signalling pathways such as NF-κB, PI3K/Akt, and MAPK, ultimately leading to reduced cancer cell proliferation, migration, and survival.^3,23,24^ Although piperidine coupled natural products like piperidine–valproic acid hybrids outperform methotrexate as anti-proliferative and cell migration inhibition,^25^ in this study, we report the synthesis of a novel series of 1-ethoxycarbonyl-3,5-bis(vanillin)–4-piperidone analogues through a two-step protocol involving aldol condensation followed by O-alkylation. These compounds were characterized and evaluated for their cytotoxic potential against human cancer cell lines (SKBR3, HepG2, DU145) and normal cells (HEK-293).

Among the synthesized derivatives, compound 4k showed significant and selective anticancer activity, making it a suitable candidate for further biological evaluation. To establish a comprehensive safety profile, we conducted a panel of *in vitro* genotoxicity and toxicity assays. The chromosomal aberration (CA) assay assesses structural chromosomal damage such as breaks and fragments, serving as a direct measure of clastogenic potential.^26^ The micronucleus (MN) assay detects both chromosomal breaks and missegregation events and is widely used to screen for genotoxicity in mammalian cells. The mitotic index (MI) provides insight into cell proliferation and cytostatic effects, with reduced indices often indicating DNA damage-induced cell cycle arrest. In addition, the erythrocyte osmotic fragility (EOF) assay was used to evaluate membrane stability under hypotonic stress, a crucial parameter for assessing the potential hemolytic toxicity of systemically active agents.^27,28^ Collectively, this study suggests the potential anticancer properties of vanillin–piperidone hybrids and establishes a preliminary genotoxic safety framework, supporting the suitability of the lead compound for further preclinical investigation.

## 2 Results and discussion

### 2.1. Chemistry

**Scheme 1:**
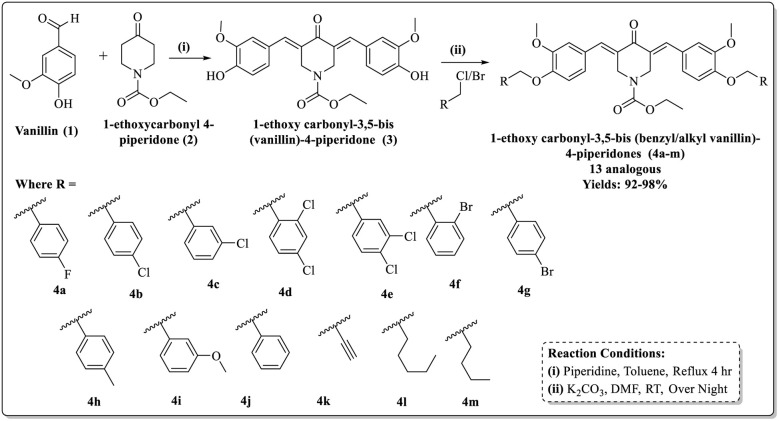
Synthesis of novel 1-ethoxy carbonyl-3,5-bis (benzyl/alkyl vanillin)–4-piperidone analogues.

The synthetic strategy adopted for the preparation of alkyl and benzyl derivatives of bis-vanillin is outlined in Scheme 1. A series of compounds (4a–4m) were synthesized in two steps. In the first step, intermediate 3 was obtained *via* an aldol condensation reaction between vanillin and 1-ethoxycarbonyl-4-piperidone in the presence of catalytic piperidine. In the second step, compound 3, which served as the common precursor for all derivatives, was subjected to O-alkylation using various aromatic and aliphatic halides in the presence of potassium carbonate to afford the final compounds 4a–4m.

All synthesized compounds were characterized by ^1^H NMR (500 MHz), ^13^C NMR (125 MHz), and high-resolution mass spectrometry (HR-MS). In the ^1^H NMR spectrum of compound 4a, successful O-benzylation was confirmed by the presence of a singlet at *δ* 5.18 ppm (s, 4H) corresponding to the benzylic methylene protons (O–CH_2_), while the methoxy protons appeared as a singlet at *δ* 3.94 ppm (s, 6H). In the ^13^C NMR spectrum, the benzylic methylene carbons and methoxy carbons were observed at *δ* 70.29 ppm (Two –CH_2_) and *δ* 56.07 ppm (Two –OCH_3_), respectively. The molecular ion peak for compound 4a was detected at *m*/*z* 656.2435 [M + H]^+^ in the HR-MS (positive mode), consistent with its calculated molecular formula. All other proton and carbon signals appeared in the expected chemical shift regions, confirming the proposed structures of the synthesized derivatives.

### 2.2. Evaluation of biological properties alkyl and benzyl derivatives of bis-vanillin

#### 2.2.1. Antidiabetic activity

The antidiabetic activity of the synthesized vanillin–piperidone analogues was evaluated through their inhibition of mammalian α-glucosidase (m-AGI) at 20 µg mL^−1^. Among the series, compounds 1, 3, 4c, and 4m emerged as the most potent inhibitors, exhibiting m-AGI inhibition values of 58.53 ± 1.03%, 57.68 ± 1.00%, 52.61 ± 1.88%, and 54.01 ± 0.36%, respectively (Table 1). These compounds also showed favourable IC_50_ values ranging from 4.27 to 4.75 µg mL^−1^, indicating their significant potential as antidiabetic agents.^29^ Although their activity was lower than that of the reference drug acarbose (89.46%, IC_50_: 2.78 µg mL^−1^), the consistent inhibition across these four compounds highlights their promise for further development. Their activity may be attributed to structural features enhancing interactions with the α-glucosidase enzyme, making them strong candidates for lead optimization in future antidiabetic drug design.

**Table 1:** Activity results of 1-ethoxy carbonyl-3,5-bis (benzyl/alkyl vanillin)–4-piperidones analogues^a^

Compound	Anti-diabetic activity	(Zone of inhibition in (mm) at 1 mg mL^−1^ at 24 h)					
		Antibacterial activity				Antifungal activity	
	m-AGI% inhibition at 20 µg mL^−1^	*E. coli*	*K. pneumoniae*	*S. aureus*	*B. subtilis*	*C. albicans*	*C. tropicalis*
1	58.53 ± 1.03 **(IC**_**50**_**: 4.27)**	7	7	8	8	13	12
3	57.68 ± 1.00 **(IC**_**50**_**: 4.33)**	NA	NA	7	NA	NA	NA
4a	30.22 ± 2.53	NA	NA	7	NA	NA	NA
4b	36.02 ± 0.26	NA	NA	7	NA	NA	NA
4c	52.61 ± 1.88 **(IC**_**50**_**: 4.75)**	NA	NA	7	NA	NA	NA
4d	40.91 ± 0.23	NA	NA	7	NA	NA	NA
4e	48.19 ± 0.52	NA	NA	7	NA	NA	NA
4f	19.23 ± 0.76	NA	NA	NA	NA	NA	NA
4g	10.19 ± 0.87	NA	NA	NA	NA	NA	NA
4h	33.03 ± 0.68	NA	NA	NA	NA	NA	NA
4i	27.16 ± 0.72	NA	NA	NA	NA	NA	NA
4j	36.55 ± 0.89	NA	NA	NA	NA	NA	NA
4k	33.31 ± 2.43	NA	NA	NA	NA	NA	NA
4l	36.65 ± 0.17	NA	NA	NA	NA	NA	NA
4m	54.01 ± 0.36 **(IC**_**50**_**: 4.62)**	NA	NA	NA	NA	NA	NA
Acarbose	**89.46 (IC**_**50**_ **: 2.78)**	**—**	**—**	**—**	**—**	**—**	**—**
Streptomycin	**—**	**24**	**24**	**24**	**24**	**—**	**—**
Ketoconozole	**—**	**—**	**—**	**—**	**—**	**24**	**24**

a Zone of inhibition (m) are mean of two independent tests; ± is the standard deviation of from three independent replicates.

#### 2.2.2. Antimicrobial activity

The antimicrobial activity of the synthesized vanillin–piperidone analogues was assessed against selected bacterial and fungal strains, with compound 1 exhibiting the most notable broad-spectrum activity (Table 1). It showed inhibition zones of 7 mm against *E. coli* and *K. pneumoniae*, 8 mm against *S. aureus* and *B. subtilis*, and antifungal activity of 13 mm and 12 mm against *Candida albicans* and *C. tropicalis*, respectively. Similar kind of broad spectrum antimicrobial properties of vanillin analogues were reported.^30^ Other compounds, such as 3 and 4a–4e, displayed only weak antibacterial activity, primarily limited to *S. aureus* (zone of inhibition: 7 mm), while the remaining analogues were largely inactive at the tested concentration. For comparison, standard antibiotics such as streptomycin and ketoconazole showed zones of 24 mm. Representative images of anti-bacterial and anti-fungal screening, are provided in the SI (Fig. S43).

#### 2.2.3. *In vitro* cytotoxicity and anticancer studies

The synthesized vanillin-based compounds were screened for their cytotoxic activity against a panel of human cell lines comprising three cancerous cell lines SKBR3 (breast), HepG2 (liver), and DU145 (prostate) and one non-cancerous line, HEK-293. Doxorubicin served as the standard reference drug demonstrated potent cytotoxicity across all cancer cell lines but with higher toxicity in HEK-293 (IC_50_ = 6.12 ± 0.5 µM). Among the tested derivatives, most of these displayed weak to moderate cytotoxicity and limited selectivity, as evidenced by high IC_50_ values in both cancerous and normal cells. However, compounds 3 and 4k exhibited the most potent and broad-spectrum anticancer activity. Compound 3 demonstrated significant cytotoxicity with IC_50_ values of 2.97 µM (SKBR3), 4.4 µM (DU145), and 20.5 µM (HEK-293) (Table 2), indicating selectivity toward malignant cells. Whereas, compound 4k showed strong activity against HepG2 (2.27 µM) and DU145 (5.01 µM), suggesting its possible anticancer properties. These values reflect marked selectivity toward hepatocellular and prostate cancer cells, with reduced activity against breast cancer and relatively moderate cytotoxicity in non-malignant cells. The present results are collaborated with earlier studies where vanillin can inhibit proliferation in various cancer cells and less toxic to non-cancerous cells.^31^ It can be assured that vanillin containing piperidone can significantly enhance its anticancer efficacy and selectivity.

**Table 2:** *In vitro* cytotoxicity and anticancer activity of 1-ethoxy carbonyl-3,5-bis (benzyl/alkyl vanillin)–4-piperidones analogues on both normal and cancer cells^a^

Compound	*In vitro* cytotoxicity						
	HEK-293	HepG2		DU145		SKBR3	
	IC_50_ (µM)	IC_50_ (µM)	TI	IC_50_ (µM)	TI	IC_50_ (µM)	TI
1	127.3 ± 8.2	391.3 ± 37.9	0.33	21.3 ± 0.8	5.98	96.5 ± 2.4	1.32
3	20.5 ± 4.9	48.4 ± 5.5	0.42	4.4 ± 0.8	4.66	2.97 ± 0.4	6.90
4a	56.1 ± 1.1	104.6 ± 1.9	0.54	72.4 ± 0.2	0.77	57.7 ± 6.5	0.97
4b	53.7 ± 3.2	112.2 ± 7.5	0.48	10.6 ± 0.05	5.07	74.6 ± 6.6	0.72
4c	264.6 ± 17.7	131.6 ± 8.1	2.01	38.3 ± 7.7	6.91	90.1 ± 3.1	2.94
4d	45.2 ± 8.5	108.9 ± 13.3	0.42	70.8 ± 4.2	0.64	68.8 ± 5.4	0.66
4e	56.7 ± 12.9	74.8 ± 3.2	0.76	45.7 ± 2.5	1.24	66.8 ± 7.3	0.85
4f	51 ± 6.5	71.05 ± 3.8	0.72	47.8 ± 3.5	1.07	82.9 ± 6.9	0.62
4g	97.3 ± 11.7	18.2 ± 0.3	5.35	85.64 ± 6.8	1.14	85 ± 6.6	1.14
4h	95.4 ± 6.6	42.4 ± 6.1	2.25	104.07 ± 11.2	0.92	89.4 ± 9.2	1.07
4i	258.2 ± 14	185.9 ± 10.9	1.39	28.09 ± 6.3	9.19	53.3 ± 8.7	4.84
4j	147.5 ± 16.6	85.6 ± 2.6	1.72	45.7 ± 9.5	3.23	37.3 ± 7.3	3.95
4k	11.5 ± 2.5	2.27 ± 1.2	5.07	5.01 ± 6.1	2.30	127.3 ± 11.7	0.09
4l	72.9 ± 8.6	129.3 ± 8.2	0.56	92.78 ± 5.5	0.79	148.2 ± 17.5	0.49
4m	125.6 ± 2.8	77.5 ± 6.4	1.62	88.5 ± 8.6	1.42	56.2 ± 6.9	2.23
Doxorubicin	6.12 ± 0.5	0.70 ± 0.5	8.74	2.5 ± 1.4	2.45	0.45 ± 0.5	13.60

a Normal cell line: HEK-293-Human embryonic kidney cell line; cancer cell lines: HepG2-Human hepatoblastoma cancer cell line; DU145-Human prostate cancer cell line; SKBR3-Human breast cancer cell line; ND = Not done; ± = is the standard deviation of mean of three independent replicates. TI = the ratio of the IC_50_ in normal cells to that in cancer cells.

##### 2.2.3.1 Structure–activity relationship (SAR) analysis

A comparison of the cytotoxicity data revealed several trends within the synthesized vanillin–piperidone series. The parent compound 3 exhibited the highest activity against DU145 and SKBR3 cells, suggesting that the free phenolic hydroxyl groups are important for maintaining cytotoxic potency. Conversion of these hydroxyl groups into O-substituted derivatives generally resulted in a reduction in activity, although the extent varied depending on the nature of the substituent. Among the halogenated benzyl derivatives (4a–4g), most compounds showed moderate to weak cytotoxicity. However, compound 4g displayed selective activity against HepG2 cells, indicating that the position and nature of the halogen substituent influence the biological response. Compounds bearing substituted benzyl groups (4h–4j) also showed only moderate activity, whereas the propargyl derivative 4k emerged as the most active analogue against HepG2 and DU145 cells. In contrast, the long-chain alkoxy derivatives 4l and 4m exhibited comparatively lower cytotoxicity. Collectively, these results suggest that the nature of the O-substituent plays an important role in modulating anticancer activity within this series, with smaller substituents being generally more favourable than bulkier aromatic or long-chain alkyl groups. The superior activity of 4k further demonstrates that subtle structural modifications around the vanillin moiety can significantly influence the biological profile of these hybrids.

#### 2.2.4. Erythrocyte osmotic fragility (EOF)

**Fig. 1:**
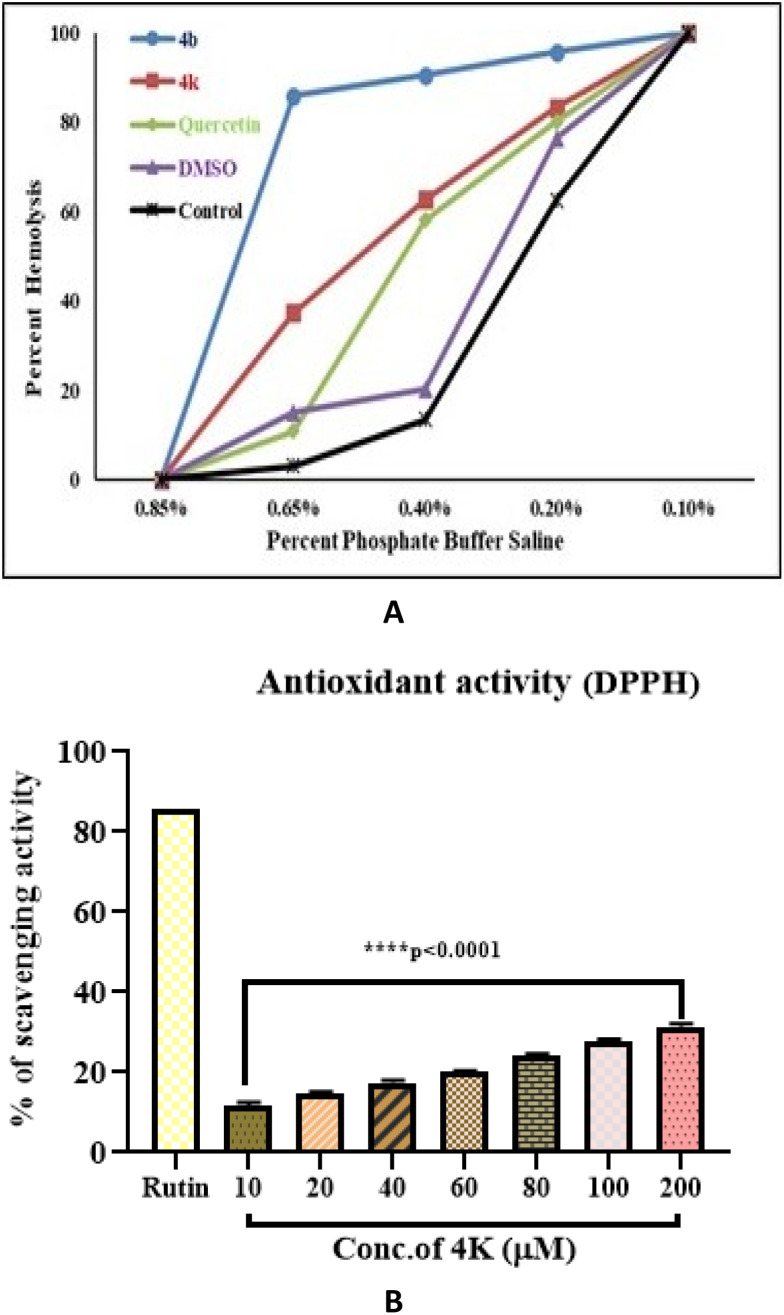
(A) Erythrocyte osmotic fragility curve of 4k; (B) percentage of free radical scavenging activity at different concentrations of 4k compared to rutin using DPPH assay.

The erythrocyte osmotic fragility assay effectively revealed how 4k interact with erythrocyte membranes under stress. As observed, peak fragility occurred at intermediate concentrations, indicating a critical threshold at which the compounds exert maximal stress on the erythrocyte membrane.^32^ The compound 4k notably reduced membrane fragility, nearly matching the efficacy of quercetin, a flavonoid with well-established antioxidant and membrane-stabilising roles (Fig. 1A). Recent studies demonstrate that quercetin protects red blood cells from oxidative damage and preserves membrane protein function in both acute and aging models and improves osmotic resistance, particularly under diabetic conditions.^33^

#### 2.2.5. Evaluation of antioxidant activity of 4k using the DPPH assay

Vanillin mostly exhibits antioxidant activity by scavenging free radicals and enhancing cellular antioxidant enzymes like SOD and catalase. Structurally modified vanillin derivatives, such as vanillin–pyrido-dipyrimidines and vanillin–taurine Schiff bases, show enhanced antioxidant potential.^34,35^ Hence, the antioxidant potential of compound 4k was assessed using the DPPH (2,2-diphenyl-1-picrylhydrazyl) radical scavenging assay, and the results are summarized in (Table 3) and illustrated in Fig. 1B. 4k exhibited a dose-dependent increase in free radical scavenging activity, ranging from 11.82% at 10 µM to 31.36% at 200 µM. Although the antioxidant potential is significantly lower compared to the reference standard Rutin, which demonstrated 85.5% scavenging activity at 100 µM. Overall, 4k demonstrates moderate free radical neutralizing activity, which may contribute partially to its biological effects but is unlikely to be its primary mode of action. Hence, it can be assumed that 4k could be acting *via* similar mechanisms, possibly by scavenging reactive oxygen species and stabilising lipid–protein interactions within the bilayer. These findings suggest that 4k is a lead candidate for further development in disorders caused by oxidative erythrocyte damage, such as diabetes and cancer.

**Table 3:** Antioxidant properties of 4k using DPPH assay^a^

4k concentrations (µΜ)	% Of antioxidant activity
200	31.36 ± 0.37****↓
100	27.84 ± 0.19****↓
80	24.33 ± 0.16****↓
60	20.08 ± 0.14****↓
40	17.49 ± 0.24****↓
20	14.78 ± 0.19****↓
10	11.82 ± 0.34****↓
**Standard: Rutin (100 µΜ)**	**85.5** ± **0.21**

a ± = is the standard deviation of mean of three independent replicates. ****↓ significate decrease from the standard (Rutin) *P* < 0.001.

#### 2.2.6. Genotoxicity evaluation of 4k in CHO-K1 cells

Considering the promising cytotoxic effects on cancer cells and moderate antidiabetic properties exhibited by 4k, it was imperative to assess its potential genotoxic effects on normal cells to establish its safety profile. For this purpose, CHO-K1 cells, were selected for *in vitro* genotoxicity testing. Based on the IC_50_ value (13.5 µM) determined in CHO-K1 cells (Table S1), three sub-lethal concentrations of 4k (2.5, 5.0, and 7.5 µM) were chosen for genetic toxicity studies and compared with Mitomycin-C (2.5 µM). Cells were treated with 4k for 24 hours, and the chromosomal aberration (CA) assay was performed to assess structural chromosomal damage and clastogenicity. The mitotic index (MI) was evaluated to monitor effects on cell division and cytostasis, while the micronucleus (MN) assay was employed to detect both clastogenic and aneugenic events. Together, all these assays provide a comprehensive evaluation of the genotoxic potential of 4k and offer valuable insight into its safety in non-malignant cellular systems.

#### 2.2.7. *In vitro* chromosomal aberration test

The *in vitro* chromosomal aberration assay was conducted using CHO-K1 cells to assess the genotoxic potential of the test compound 4k. Cells were exposed to 4k at concentrations of 2.5, 5.0, and 7.5 µM, and chromosomal aberrations were evaluated in 300 metaphase spreads per treatment group. The vehicle control group (1% DMSO) exhibited 9.23% aberrant metaphases. Treatment with 4k induced a concentration-dependent, but statistically non-significant, increase in aberrant metaphases compared to the control (*p* > 0.05). The highest frequency observed was 10.33% at 7.5 µM. In contrast, the positive control Mitomycin-C (2.5 µM) significantly increased the percentage of aberrant metaphases to 46.7 ± 13% (*p* < 0.001), compared to vehicle control (Table 5).

**Fig. 2:**
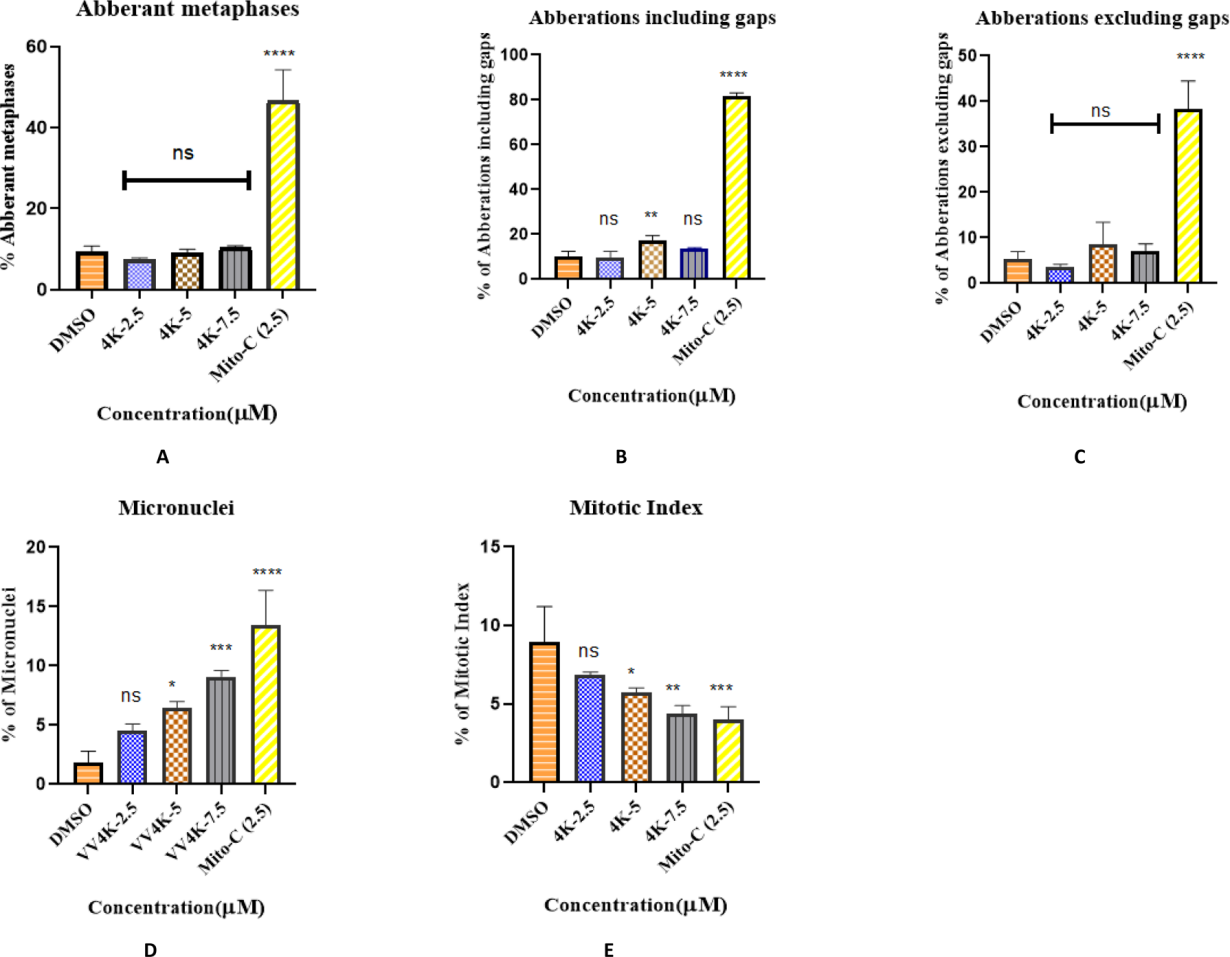
Genotoxicity studies of 4k at different concentration in CHO-K1 cells. (A). Aberrant metaphase (B). Total number of aberrations including gaps (C). Total number of aberrations excluding gaps (D). Induction of micronucleus (E). Changes in mitotic index. *P < 0.05; ** = *P* < 0.01; **** = *P* < 0.001; ns = not significant (one-way analysis of variance followed by Dunnet test).

While comparing the percentage of total chromosomal aberrations including gaps, and aberrations excluding gaps, the DMSO control group exhibited aberrations (10.2% including gaps; 5.42% excluding gaps) whereas, the positive control Mitomycin-C caused a significant increase in chromosomal aberrations (81.7% including gaps and 38.3% excluding gaps, *p* < 0.001). Treatment with 4k resulted in slight, concentration-dependent increases in aberrations. The aberrations primarily included chromatid and chromosome breaks, fragments, and minutes, with no occurrence of severe lesions such as dicentric chromosomes or translocations. At 7.5 µM, 4k induced 13.7 ± 2.07% aberrations including gaps and 7.00% excluding gaps (Table 4 & Fig. 2B and C), which were not statistically significant compared to the control (*p* > 0.05), indicating a lack of clastogenicity. Overall, chromosome aberration study confirm that 4k does not significantly induce clastogenic effect, supporting its genotoxic safety.

**Table 4:** Induction of percentage of chromosomal aberrations at different concentrations of 4k in CHO-K1 cells at 24 h of post-treatment^a^

Chemicals	Dose (µM)	No. of metaphase spreads observed	No. of aberrant metaphase spreads	Types and number of aberrations								% Of aberrant metaphases	% Of aberrations (including gaps)	% Of aberrations (excluding gaps)
				Ct		Cm		F	m	T	endo			
				G	B	G	B							
DMSO	1%	315	29	15	15	0	1	0	0	0	0	9.23 ± 0.92	10.2 ± 1.37	5.42 ± 0.91
4k	2.5	300	22	8	9	5	1	0	0	0	0	7.33 ± 0.65 ^ns^	9.67 ± 0.32 ^ns^	3.67 ± 0.59 ^ns^
	5	300	27	10	8	8	9	0	0	0	0	9.00 ± 0.07 ^ns^	17.3 ± 4.26 **	8.67 ± 1.10 ^ns^
	7.5	300	31	12	15	4	3	0	0	0	0	10.33 ± 0.38 ^ns^	13.7 ± 2.07 ^ns^	7.00 ± 0.53 ^ns^
Mitomycin-C	2.5	300	140	104	43	13	8	7	7	5	16	46.7 ± 13****	81.7 ± 42.8****	38.3 ± 11.1****

a ± = SEM (standard error mean from three replicates); Ctd = chromatid; G = gap; B = breaks; Cm = chromosome; F = fragment; m = minute; dic = dicentric; T = translocations; endo: endoduplication; DMSO = vehicle control; Mitomycin-C = positive control; significant at level compared to DMSO ** = *P* < 0.01; **** = *P* < 0.001; ns = not significant (Dunnet's multiple comparison test).

#### 2.2.8. *In vitro* micronucleus assay

The aneugeic potential of compound 4k was evaluated using the *in vitro* micronucleus (MN) assay in CHO-K1 cells. Cells were treated with three sub-lethal concentrations of 4k (2.5, 5, and 7.5 µM) for 24 hours, and MN formation was quantified relative to the vehicle control (1% DMSO). The vehicle control group exhibited a low baseline frequency of MN (1.85%), confirming minimal genotoxicity. Treatment with 4k resulted in a dose-dependent increase in MN formation. At 2.5 µM, a slight, non-significant increase was observed (4.52%, ns), while at 5 µM, a statistically significant rise occurred (6.44%, *p* < 0.05) (Table 5 & Fig. 2D). The effect was more pronounced at 7.5 µM, where MN frequency reached 9.09% (*p* < 0.001). The positive control, Mitomycin-C induced a higher frequency of MN 13.41% (*p* < 0.0001). These results demonstrate that 4k induces micronucleus formation in a concentration-dependent manner, indicating mild to moderate genotoxicity, especially at higher concentrations.

**Table 5:** Induction of micronuclei at different concentrations of 4k in CHO-K1 cells at 24 h of post-treatment^a^

Test chemicals	Concentration (µM)	Total number of binucleated cells scored	Number of micro-nucleated cells	Total number of micronuclei	Percentage of micronuclei/1000 cells
DMSO (vehicle control)	1%	4809	8	8	1.85 ± 0.553
4k	2.5	3099	14	14	4.52 ± 2.23^ns^
	5	3101	20	20	6.44 ± 3.84*
	7.5	3076	28	28	9.09 ± 6.06***
Mitomycin-C	2.5	9231	108	122	13.41 ± 9.6****

a ± = SEM (standard error mean from three replicates); DMSO = vehicle control; Mitomycin-C = positive control; significant at level compared to DMSO *P < 0.05; *** = *P* < 0.001; **** = *P* < 0.0001; ns = not significant (Dunnet's multiple comparison test).

#### 2.2.9. *In vitro* mitotic index assay

The mitotic index assay is a valuable tool for evaluating the proportion of cells undergoing mitosis in each population, often used to assess the effects of various chemicals on cell division. In the vehicle control, using DMSO at a 1% concentration, displayed the highest mitotic index at 8.99%, with 299 dividing cells observed out of a total of 3374 whereas, Mitomycin-C, used as a reference compound at 2.5 µM, showed the lowest mitotic index of 3.99 ± 5.56, with 145 dividing cells observed from a total of 3674 (Table 6 & Fig. 2). All the three concentrations of 4k induced a dose-dependent decrease in the percentage of mitotic index. At a concentration of 2.5 µM, 4k showed a mitotic index of 6.86%, with 215 dividing cells out of 3136. As the concentration increased to 5 µM, the mitotic index dropped further to 5.75% with 175 dividing cells among 3043, and at the highest concentration of 7.5 µM, the mitotic index was reduced to 4.39% with 137 dividing cells out of 3125. From this study, it can be assumed that the inhibitory effects of 4k on mitotic activity as its concentration increases. This decrease in the mitotic division could be due to the clastogenic effects of 4k either at G1 or S phase or delayed of DNA repair ultimately reflects the lesser number of cells at M phase of the cell cycle.

**Table 6:** Percentage of mitotic index of at different concentrations of 4k in CHO-K1 cells at 24 h of post-treatment^a^

Test chemicals	Concentration (µM)	Total number of cells	Total number of dividing cells observed	Percentage of mitotic index
DMSO (vehicle control)	1%	3374	299	8.99 ± 0.547
4k	2.5	3136	215	6.86 ± 2.37 ^ns^
	5	3043	175	5.75 ± 3.60*
	7.5	3125	137	4.39 ± 5.12**
Mitomycin-C	2.5	3674	145	3.99 ± 5.56***

a ± = SEM (standard error mean from three replicates); DMSO = vehicle control; Mitomycin-C = positive control; significant at level compared to DMSO *P < 0.05; ** = *P* < 0.01; *** = *P* < 0.001; ns = not significant (Dunnet's Multiple Comparison Test).

#### 2.2.10. Cell cycle analysis in normal and cancer cells

**Fig. 3:**
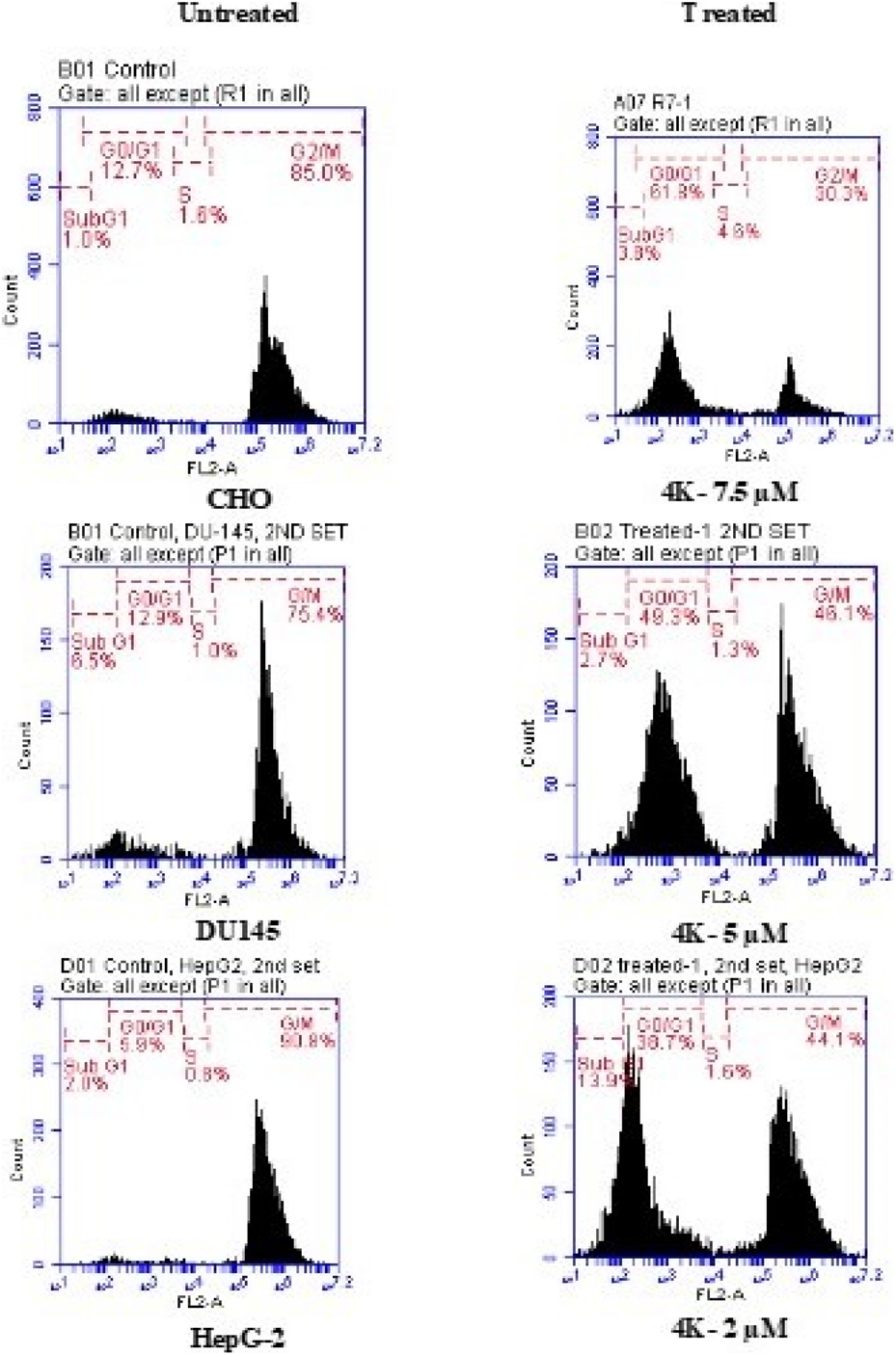
Flow cytometric analysis of cell cycle progression in normal (CHO-K1) and cancer cells (DU-145 & HepG2) treated with IC_50_ concentration of 4k.

The effect of 4k on cell cycle distribution was assessed in normal CHO-K1 cells and two human cancer cell lines, DU145 (prostate cancer) and HepG2 (hepatocellular carcinoma), using flow cytometry. Cells were treated with respective IC_50_ concentrations of 4k for 24 hours, and the percentage of cells in the Sub-G1, G0/G1, S, and G2/M phases were quantified (Table 7 & Fig. 3). In untreated CHO-K1 cells, a majority of the population (85.0%) was observed in the G2/M phase, consistent with active proliferation. Upon treatment with 7.5 µM 4k, a significant redistribution of cells to the G0/G1 phase (61.8%) was observed, accompanied by a reduction in G2/M phase cells to 30.3%, suggesting G0/G1 phase arrest in normal cells. A modest increase in Sub-G1 content (1.0 to 3.8%) indicated minimal apoptosis. In DU145 prostate cancer cells, untreated populations exhibited a predominance in G2/M (75.4%) and G0/G1 (12.9%) phases. Following treatment with 5 µM 4k, a shift toward G0/G1 phase (49.3%) was observed, with a concurrent decrease in G2/M (46.1%) and Sub-G1 (6.5 to 2.7%) populations. This suggests cell cycle arrest at G0/G1, with no significant induction of apoptosis in this cell line. In contrast, HepG2 cells responded more dramatically to treatment with 2 µM 4k. The Sub-G1 population, indicative of apoptotic cells, increased markedly from 2.0 to 13.9%, while G2/M phase cells decreased from 90.8 to 44.1%. A marked increase in G0/G1 phase cells (from 5.9 to 38.7%) was also observed, suggesting a combined effect of cell cycle arrest and apoptosis induction. However, detailed studies are required to confirm this toxic effects. Collectively, these findings demonstrate that 4k induces G0/G1 phase arrest in both normal and cancerous cell lines but elicits a more pronounced cell cycle arrest in HepG2 cells, correlated with its cytotoxicity results in liver cancer. The differential response between cancer and normal cells supports the selective anticancer potential of 4k and warrants further mechanistic investigations.

**Table 7:** Compound 4k-induced changes in cell cycle phase distribution in CHO-K1, DU145, and HepG2 cells

Cell cycle phases	CHO-K1 untreated/treated	DU145 untreated/treated	HepG2 untreated/treated
Sub-G1 (%)	1.0/3.8	6.5/2.7	2.0/13.9
G0/G1 (%)	12.7/61.8	12.9/49.3	5.9/38.7
S (%)	1.6/4.6	1.0/1.3	0.8/1.6
G2/M (%)	85.0/30.3	75.4/46.1	90.8/44.1

Percentage of cells in different phases of the cell cycle post 24 h treatment with 4k (treated) comparison to untreated cells.

So, based on *in vitro* genotoxicity studies, it is evident that 4k exhibits genotoxic potential, particularly at higher concentrations by increasing the induction of aberrant metaphases (clastogenic), micronucleus (aneugenic) and reducing the rate of cell division (mitotoxic). Whereas, at lower concentrations were found to be non-genotoxic in normal CHO-K1 cells. Earlier studies have also reported that vanillin itself can be mutagenic at higher concentrations as it induces sister chromatid exchange and chromosomal aberrations in human lymphocytes.^36^ Further, it has been reported that vanillin-induced cytotoxic effects in liver cells may result from its CYP3A-mediated metabolism, which leads to the formation of quinone-like intermediates that covalently bind to glutathione.^37^ Hence, it can be assumed that vanillin and its derivatives may exhibit either mutagenic or anti-mutagenic properties, depending on their concentration and the physiological context. At lower concentrations, vanillin has been shown to possess anti-mutagenic and antioxidant activities, effectively reducing DNA damage induced by known mutagens in various *in vitro* and *in vivo* models. Therefore, the dual nature of vanillin derivatives as both protective and potentiate nature can be influenced by metabolic activation pathways and cellular context.

## 3 Experimental

### 3.1. Chemistry

All chemicals and reagents were procured from Aldrich (India) and AVRA Chemicals Pvt. Ltd (India) and were used as received without further purification. Thin-layer chromatography (TLC) was performed using silica gel 60 F254 plates (Merck, Germany), and the developed spots were visualized under UV light or by staining with 5% H_2_SO_4_ in methanol. Melting points were determined using an A. KRUSS Optronic melting point apparatus (Germany) and are reported without correction. ^1^H NMR (500 MHz) and ^13^C NMR (125 MHz) spectra were recorded at room temperature in CDCl_3_ on a Bruker Avance-III 500 MHz NMR spectrometer (Switzerland). Chemical shifts (*δ*) are reported in parts per million (ppm) relative to tetramethylsilane (TMS) as the internal standard, with CDCl_3_ as solvent (*δ*_H = 7.26 ppm; *δ*_C = 77.0 ppm). NMR data are presented as: chemical shift (*δ*), multiplicity (s = singlet, d = doublet, t = triplet, dd = doublet of doublets, q = quartet, br = broad, m = multiplet), coupling constants (J in Hz), and integration values.

#### 3.1.1. General procedure for synthesis of 1-ethoxy carbonyl-3,5-bis (benzyl/alkyl vanillin)–4-piperidone analogues

##### 3.1.1.1 Synthesis of 1-ethoxy carbonyl-3,5-bis (vanillin)–4-piperidone (3)

A mixture of vanillin 1 (1.779 g, 11.69 mmol), 1-ethoxycarbonyl-4-piperidone 2 (1.000 g, 5.84 mmol), and a catalytic amount of piperidine was placed in a round-bottom flask. To this, 10 mL of toluene was added, and the reaction mixture was refluxed for 4–8 h using a Dean–Stark apparatus to continuously remove water. After completion, the reaction mixture was cooled to room temperature, and the resulting pale-yellow solid was filtered, washed repeatedly with distilled water followed by methanol, and dried to afford 1-ethoxycarbonyl-3,5-bis(vanillin)–4-piperidone (3) in pure form (Yield: 95%).

###### 3.1.1.1.1 Ethyl 3,5-bis((*E*)-4-hydroxy-3-methoxybenzylidene)-4-oxopiperidine-1-carboxylate (3)

Pale yellow solid, yield: 97%, m.p. 194–196 °C; ^1^H-NMR (500 MHz, CDCl_3_) *δ* (ppm): 8.69 (s, 2H), 7.71 (s, 2H), 6.97 (s, 6H), 4.79 (s, 4H), 4.08 (m, 2H), 3.89 (d, *J* = 19.4 Hz, 6H), 1.15 (m, 3H) (Fig. S1); ^13^C-NMR (125 MHz, CDCl_3_) *δ* (ppm): 186.40, 155.07, 148.25, 147.42, 137.29, 129.40, 124.53, 115.56, 114.10, 61.59, 55.83, 45.83, 14.38 (Fig. S2); HR-MS found [M + H]^+^ 440.1704 calculated [M] for C_24_H_25_NO_7_ is 439.4640 (Fig. S3).

##### 3.1.1.2 Synthesis of 1-ethoxy carbonyl-3,5-bis (benzyl/alkyl vanillin)–4-piperidones (4a–m)

A solution of 1-ethoxycarbonyl-3,5-bis(vanillin)–4-piperidone (3) (0.500 g, 1.14 mmol) and potassium carbonate (0.314 g, 1.59 mmol) in 15 mL of dry *N*,*N*-dimethylformamide (DMF) was stirred at room temperature. To this mixture, benzyl bromide/chloride or the appropriate alkyl halide (1.00 mmol) was added dropwise with continuous stirring. The reaction mixture was stirred overnight at ambient temperature. Upon completion, the mixture was poured into 500 mL of cold water with vigorous stirring. The resulting precipitate was filtered, washed with water, and dried under vacuum to yield the corresponding O-alkylated products (4a–4m) as pale-yellow solids in excellent yields (92–98%). The above synthetic procedures were adopted from previously reported literature.^38^

##### 3.1.1.3 Spectral data of synthesized compounds

###### 3.1.1.3.1 Ethyl 3,5-bis((*E*)-4-((4-fluorobenzyl)oxy)-3-methoxybenzylidene)-4-oxopiperidine-1-carboxylate (4a)

Pale yellow solid, yield: 97%, m.p. 171–173 °C, ^1^H NMR (500 MHz, CDCl_3_) *δ* 7.77 (s, 2H), 7.44 (dd, *J* = 8.6, 5.4 Hz, 4H), 7.11 (m, 4H), 6.98 (dd, *J* = 33.3, 7.3 Hz, 6H), 5.18 (s, 4H), 4.80 (s, 4H), 4.10 (t, *J* = 7.1 Hz, 2H), 3.94 (s, 6H), 1.16 (t, *J* = 7.1 Hz, 3H) (Fig. S4); ^13^C NMR (125 MHz, CDCl_3_): *δ* (ppm) 186.74, 163.54, 161.58, 155.27, 149.52, 149.26, 132.34, 132.32, 129.21, 129.15, 128.38, 123.88, 115.67, 115.50, 114.34, 113.57, 70.29, 61.92, 56.07, 45.12, 14.53 (Fig. S5); HR-MS found M + H, 656.2435. Calculated for C_38_H_35_F_2_NO_7_: M, 655.6948 (Fig. S6).

###### 3.1.1.3.2 Ethyl 3,5-bis((*E*)-4-((3-chlorobenzyl)oxy)-3-methoxybenzylidene)-4-oxopiperidine-1-carboxylate (4b)

Pale yellow solid, yield: 95%, m.p. 189–191 °C, ^1^H-NMR (500 MHz, CDCl_3_) *δ* 7.76 (s, 2H), 7.41 (m, 8H), 7.01 (d, *J* = 9.2 Hz, 4H), 6.92 (d, *J* = 8.1 Hz, 2H), 5.18 (s, 4H), 4.79 (s, 4H), 4.11 (q, *J* = 7.1 Hz, 2H), 3.94 (s, 6H), 1.16 (t, *J* = 7.1 Hz, 3H) (Fig. S7); ^13^C NMR (125 MHz, CDCl_3_): *δ* (ppm) 186.73, 155.26, 149.51, 149.15, 135.11, 133.88, 130.44, 128.85, 128.60, 128.45, 123.86, 114.36, 113.57, 70.17, 61.93, 56.08, 45.11, 14.53 (Fig. S8); HR-MS found M^+^, 688.1831. Calculated for C_38_H_35_Cl_2_NO_7_: M, 688.5980 (Fig. S9).

###### 3.1.1.3.3 Ethyl 3,5-bis((*E*)-4-((4-chlorobenzyl)oxy)-3-methoxybenzylidene)-4-oxopiperidine-1-carboxylate (4c)

Pale yellow solid, yield: 98%, m.p. 179–181 °C, ^1^H NMR (500 MHz, CDCl_3_) *δ* 7.77 (s, 2H), 7.48 (s, 2H), 7.33 (m, 6H), 7.02 (d, *J* = 9.3 Hz, 4H), 6.93 (d, *J* = 8.1 Hz, 2H), 5.19 (s, 4H), 4.80 (s, 4H), 4.11 (d, *J* = 7.1 Hz, 2H), 3.96 (s, 6H), 1.16 (t, *J* = 7.1 Hz, 3H) (Fig. S10); ^13^C NMR (125 MHz, CDCl_3_): *δ* (ppm) 186.75, 155.27, 149.53, 149.12, 138.70, 134.62, 129.96, 128.54, 128.23, 127.28, 125.22, 114.37, 113.61, 70.17, 61.93, 56.09, 45.11, 14.53 (Fig. S11); HR-MS found M^+^, 688.1778. Calculated for C_38_H_35_Cl_2_NO_7_: M, 688.5980 (Fig. S12).

###### 3.1.1.3.4 Ethyl 3,5-bis((*E*)-4-((2,4-dichlorobenzyl)oxy)-3-methoxybenzylidene)-4-oxopiperidine-1-carboxylate (4d)

Pale yellow solid, yield: 96%, m.p. 167–169 °C, ^1^H NMR (500 MHz, CDCl_3_) *δ* 7.76 (s, 1H), 7.58 (d, *J* = 1.7 Hz, 2H), 7.47 (d, *J* = 8.2 Hz, 2H), 7.31 (m, 2H), 7.01 (d, *J* = 9.5 Hz, 4H), 6.91 (d, *J* = 8.1 Hz, 2H), 5.15 (s, 4H), 4.79 (s, 4H), 4.10 (d, *J* = 7.1 Hz, 2H), 3.95 (s, 6H), 1.16 (t, *J* = 7.1 Hz, 3H) (Fig. S13); ^13^C NMR (125 MHz, CDCl_3_): *δ* (ppm) 186.71, 155.26, 149.54, 148.86, 136.90, 132.84, 132.11, 130.67, 129.14, 128.73, 126.45, 123.79, 114.36, 113.67, 69.59, 61.95, 56.06, 45.10, 14.53 (Fig. S14); HR-MS found M + H, 758.1032. Calculated for C_38_H_33_Cl_4_NO_7_: M, 757.4820 (Fig. S15).

###### 3.1.1.3.5 Ethyl 3,5-bis((*E*)-4-((3,4-dichlorobenzyl)oxy)-3-methoxybenzylidene)-4-oxopiperidine-1-carboxylate (4e)

Pale yellow solid, yield: 97%, m.p. 156–158 °C, ^1^H NMR (500 MHz, CDCl_3_) *δ* 7.74 (s, 2H), 7.56 (d, *J* = 1.9 Hz, 2H), 7.45 (d, *J* = 8.2 Hz, 2H), 7.29 (m, 2H), 6.99 (d, *J* = 9.5 Hz, 3H), 6.89 (d, *J* = 8.1 Hz, 2H), 5.13 (s, 4H), 4.77 (s, 4H), 4.08 (q, *J* = 7.1 Hz, 2H), 3.93 (s, 6H), 1.14 (t, *J* = 7.1 Hz, 3H) (Fig. S16); ^13^C NMR (125 MHz, CDCl_3_): *δ* (ppm) 186.70, 155.26, 149.54, 148.86, 136.90, 132.84, 132.10, 130.67, 129.14, 128.73, 126.45, 123.80, 114.37, 113.67, 69.59, 61.95, 56.06, 45.10, 14.53 (Fig. S17); HR-MS found M + H, 758.0995. Calculated for C_38_H_33_Cl_4_NO_7_: M, 757.4820 (Fig. S18).

###### 3.1.1.3.6 Ethyl 3,5-bis((*E*)-4-((2-bromobenzyl)oxy)-3-methoxybenzylidene)-4-oxopiperidine-1-carboxylate (4f)

Pale yellow solid, yield: 95%, m.p. 171–173 °C, ^1^H NMR (500 MHz, CDCl_3_) *δ* 7.77 (s, 2H), 7.60 (m, 4H), 7.35 (td, *J* = 7.6, 1.0 Hz, 2H), 7.21 (td, *J* = 7.8, 1.5 Hz, 2H), 7.03 (d, *J* = 11.5 Hz, 4H), 6.93 (d, *J* = 8.2 Hz, 2H), 5.29 (s, 4H), 4.80 (s, 4H), 4.11 (q, *J* = 7.1 Hz, 2H), 3.97 (s, 6H), 1.17 (t, *J* = 7.1 Hz, 3H) (Fig. S19); ^13^C NMR (125 MHz, CDCl_3_): *δ* (ppm) 186.77, 155.27, 149.48, 149.08, 135.85, 132.61, 129.29, 128.63, 128.49, 127.69, 121.93, 114.49, 113.55, 77.28, 77.03, 76.78, 70.20, 61.92, 56.18, 45.13, 14.55 (Fig. S20); HR-MS found M + H, 778.0785. Calculated for C_38_H_35_Br_2_NO_7_: M, 777.5060 (Fig. S21).

###### 3.1.1.3.7 Ethyl 3,5-bis((*E*)-4-((4-bromobenzyl)oxy)-3-methoxybenzylidene)-4-oxopiperidine-1-carboxylate (4g)

Pale yellow solid, yield: 95%, m.p. 159–161 °C, ^1^H-NMR (400 MHz, CDCl_3_): *δ* (ppm) 7.74 (s, 2H), 7.51 (d, *J* = 8.4 Hz, 5H), 7.32 (d, *J* = 8.3 Hz, 3H), 6.98 (d, *J* = 8.3 Hz, 4H), 6.89 (d, *J* = 8.1 Hz, 2H), 5.15 (s, 4H), 4.77 (s, 4H), 4.08 (d, *J* = 7.1 Hz, 2H), 3.92 (d, *J* = 4.7 Hz, 6H), 1.14 (m, 3H) (Fig. S22). ^13^C NMR (125 MHz, CDCl_3_) (500 MHz, CDCl_3_) *δ* (ppm) 191.15, 159.87, 154.19, 153.85, 141.72, 140.51, 136.39, 135.28, 133.86, 133.08, 128.68, 126.54, 119.07, 118.40, 74.78, 66.54, 60.80, 49.86, 19.28 (Fig. S23). HR-MS found M + H, 778.0793. Calculated for C_38_H_35_Br_2_NO_7_: M, 777.5060 (Fig. S24).

###### 3.1.1.3.8 Ethyl 3,5-bis((*E*)-3-methoxy-4-((4-methylbenzyl)oxy)benzylidene)-4-oxopiperidine-1-carboxylate (4h)

Pale yellow solid, yield: 92%, m.p. 173–175 °C, ^1^H NMR (500 MHz, CDCl_3_) *δ* 7.76 (s, 2H), 7.38 (m, 4H), 7.20 (d, *J* = 7.9 Hz, 4H), 6.97 (dd, *J* = 25.7, 8.1 Hz, 6H), 5.19 (s, 4H), 4.79 (s, 4H), 4.10 (d, *J* = 7.1 Hz, 2H), 3.94 (s, 6H), 2.37 (s, 6H), 1.16 (t, *J* = 7.1 Hz, 3H) (Fig. S25); ^13^C NMR (125 MHz, CDCl_3_): *δ* (ppm) 186.75, 155.28, 149.54, 149.45, 137.79, 133.55, 130.25, 129.33, 128.07, 127.32, 123.99, 114.35, 113.47, 70.80, 61.89, 56.09, 45.13, 21.21, 14.54 (Fig. S26); HR-MS found M + H, 648.2948. Calculated for C_40_H_41_NO_7_: M, 647.7680 (Fig. S27).

###### 3.1.1.3.9 Ethyl 3,5-bis((*E*)-3-methoxy-4-((3-methoxybenzyl)oxy)benzylidene)-4-oxopiperidine-1-carboxylate (4i)

Pale yellow solid, yield: 90%, m.p. 171–173 °C, ^1^H NMR (500 MHz, CDCl_3_) *δ* 7.29 (m, 4H), 7.05 (m, 8H), 6.89 (m, 4H), 5.20 (d, *J* = 12.0 Hz, 4H), 4.79 (s, 4H), 4.10 (d, *J* = 7.1 Hz, 2H), 3.94 (d, *J* = 9.2 Hz, 6H), 3.82 (d, *J* = 11.6 Hz, 6H), 1.16 (t, *J* = 7.1 Hz, 2H) (Fig. S28); ^13^C NMR (125 MHz, CDCl_3_): *δ* (ppm) 186.83, 159.95, 155.34, 149.44, 138.28, 130.34, 129.81, 128.22, 124.02, 119.41, 114.32, 113.58, 113.47, 112.70, 70.80, 61.59, 56.13, 55.38, 45.18, 14.62 (Fig. S29). HR-MS found M + H, 680.2830. Calculated for C_40_H_41_NO_9_: M, 679.7660 (Fig. S30).

###### 3.1.1.3.10 Ethyl 3,5-bis((*E*)-4-(benzyloxy)-3-methoxybenzylidene)-4-oxopiperidine-1-carboxylate (4j)

Pale yellow solid, yield: 96%, m.p. 174–176 °C, ^1^H NMR (500 MHz, CDCl_3_) *δ* 7.77 (s, 2H), 7.47 (d, *J* = 7.3 Hz, 4H), 7.41 (dd, *J* = 10.2, 4.7 Hz, 4H), 7.35 (d, *J* = 7.3 Hz, 2H), 6.98 (dd, *J* = 27.1, 8.6 Hz, 6H), 5.23 (s, 4H), 4.80 (s, 4H), 4.10 (t, *J* = 7.1 Hz, 2H), 3.95 (s, 6H), 1.16 (t, *J* = 7.1 Hz, 3H) (Fig. S31); ^13^C NMR (125 MHz, CDCl_3_): *δ* (ppm) 186.76, 155.28, 149.49, 136.61, 130.33, 128.66, 128.04, 127.23, 121.97, 114.38, 113.52, 70.90, 61.90, 56.11, 45.13, 14.55 (Fig. S32). HR-MS found M + H, 620.2655. Calculated for C_38_H_37_NO_7_: M, 619.7140 (Fig. S33).

###### 3.1.1.3.11 Ethyl 3,5-bis((*E*)-3-methoxy-4-(prop-2-yn-1-yloxy)benzylidene)-4-oxopiperidine-1-carboxylate (4k)

Pale yellow solid, yield: 90%, m.p. 144–146 °C, ^1^H NMR (500 MHz, CDCl_3_) *δ* 7.77 (s, 2H), 7.11 (m, 6H), 4.83 (m, 8H), 4.11 (d, *J* = 7.1 Hz, 2H), 3.93 (s, 6H), 2.57 (t, *J* = 2.4 Hz, 2H), 1.17 (t, *J* = 7.1 Hz, 3H) 7140 (Fig. S34); ^13^C NMR (125 MHz, CDCl_3_): *δ* (ppm) 186.74, 155.27, 149.47, 147.99, 130.63, 128.92, 123.65, 114.22, 113.71, 78.04, 77.31, 77.06, 76.81, 76.29, 61.94, 56.63, 56.01, 45.11, 14.54 7140 (Fig. S35); HR-MS found M + H, 516.2009. Calculated for C_30_H_29_NO_7_: M, 515.5620 7140 (Fig. S36).

###### 3.1.1.3.12 Ethyl 3,5-bis((*E*)-4-(hexyloxy)-3-methoxybenzylidene)-4-oxopiperidine-1-carboxylate (4l)

Pale yellow solid, yield: 95%, m.p. 154–156 °C, ^1^H NMR (500 MHz, CDCl_3_) *δ* 7.69 (s, 2H), 7.03 (m, 8H), 4.78 (s, 4H), 4.05 (d, *J* = 2.8 Hz, 4H), 3.88 (d, *J* = 6.1 Hz, 6H), 1.83 (d, *J* = 3.6 Hz, 4H), 1.40 (d, *J* = 60.9 Hz, 12H), 1.13 (dd, *J* = 8.4, 5.3 Hz, 3H), 0.91 (s, 6H) 7140 (Fig. S37); ^13^C NMR (125 MHz, CDCl_3_): *δ* (ppm) 185.13, 153.94, 148.85, 148.02, 135.83, 129.11, 126.36, 123.15, 113.25, 111.54, 67.75, 60.55, 54.94, 44.01, 30.33, 27.87, 24.42, 21.35, 13.41, 12.94 7140 (Fig. S38); HR-MS found M + H, 608.3576. Calculated for C_36_H_49_NO_7_: M, 607.7880 7140 (Fig. S39).

###### 3.1.1.3.13 Ethyl 3,5-bis((*E*)-3-methoxy-4-(pentyloxy)benzylidene)-4-oxopiperidine-1-carboxylate (4m)

Pale yellow solid, yield: 95%, m.p. 149–151 °C, ^1^H NMR (500 MHz, CDCl_3_) *δ* 7.77 (s, 2H), 7.00 (m, 6H), 4.82 (s, 4H), 4.10 (m, 6H), 3.92 (s, 6H), 1.90 (d, *J* = 7.1 Hz, 4H), 1.48 (m, 8H), 1.17 (t, *J* = 7.1 Hz, 3H), 0.96 (t, *J* = 7.2 Hz, 6H) 7140 (Fig. S40); ^13^C NMR (125 MHz, CDCl_3_): *δ* (ppm) 186.78, 155.31, 150.01, 149.23, 130.09, 127.64, 124.20, 114.32, 112.45, 69.02, 61.87, 56.11, 45.15, 28.77, 28.08, 22.45, 14.54, 13.99 7140 (Fig. S41); HR-MS found M + H, 580.3260. Calculated for C_34_H_45_NO_7_: M, 579.7340 7140 (Fig. S42).

### 3.2. Erythrocyte osmotic fragility

Erythrocyte fragility was assessed using heparinized human blood subjected to hypotonic phosphate-buffered saline (PBS, 10% stock) solutions with progressively decreasing concentrations, ranging from 0.85 to 0.10%.^27,28^ Test compounds (1 µL) were added to the blood samples from the stock of 10 mg mL^−1^, with quercetin as a positive control and DMSO as a negative control. The samples were incubated at 37 °C for 30 min to allow interaction with the test compounds. Following incubation, the treated cells were exposed to a series of hypotonic PBS solutions (0.85%, 0.65%, 0.4%, 0.2%, and 0.1%) and further incubated under the same conditions for 30 min with gentle agitation. Afterwards, the cell suspensions were centrifuged at 3000 rpm for 5 min at 4 °C to separate the supernatant. The degree of haemolysis was then quantified spectrophotometrically by measuring absorbance at 540 nm. The percentage of haemolysis was determined by calculating the ratio of the optical density (OD) of the supernatant obtained from each PBS concentration to the OD of a standard representing 100% haemolysis. Osmotic fragility curves were generated by plotting the haemolysis percentage against PBS concentration, and the mean erythrocyte fragility (MEF_50_) was computed using Table Curve 2D Windows v4.07 (AISN Software Inc., USA).

### 3.3. α-Glucosidase enzyme inhibitory activity

The enzyme Inhibitory activity of the 1-ethoxy carbonyl-3,5-bis (benzyl/alkyl vanillin)–4-piperidones were measured by using α-glucosidase Inhibitory Assay.^39^ The m-AGI buffer was prepared by 1.77 g of dipotassium hydrogen phosphate was weighed and dissolved in 100 mL of distilled water, pH was adjusted to 6.8. The enzyme was prepared by the 1.0 g of rat intestinal acetone powder dissolved in 10 mL normal saline done vortex and sonification 3–4 min under the ice. Then centrifugation was done with 7500 rpm for 30 min and then the enzyme was stored in the freeze. The substrate was prepared with 75.32 mg of substrate (4-nitrophenyl a-d-glucopyranoside 99%) and to this 10 mL of distilled was added. Also, the azide 0.04 mg was added to enzyme preparation. The samples were prepared by 2 mg of sample was taken in 1 mL vial and added. 1 mL of DMSO. In addition, from this standard solution further 7 dilutions were made. Rat intestinal acetone powder in normal saline (100 : 1; W/V) was sonicated properly and the supernatant was used as a source of crude intestinal α-glucosidase. In brief, 20 µL of test sample [2 mg mL^−1^ dimethyl sulphoxide (DMSO) solution] were reconstituted in 100 µL of 100 mM-phosphate buffer (pH 6.8) in 96 well microplate and incubated at 26 °C with 50 µL of substrate [5 mM, *p*-nitro phenyl-α-glucosidase (*p*-NPG) prepared in same buffer] was added.

### 3.4. Antimicrobial assay

Antimicrobial activity was evaluated against six microbial strains: two Gram-negative bacteria (*Escherichia coli* MTCC 730 and *Klebsiella pneumoniae* MTCC 109), two Gram-positive bacteria (*Staphylococcus aureus* MTCC 96 and *Bacillus subtilis* MTCC 121), and two fungal strains (*Candida albicans* MTCC 227 and *Candida tropicalis* MTCC 230). All strains were obtained from the Microbial Type Culture Collection and Gene Bank (MTCC), Chandigarh. Bacterial strains were cultured on Mueller–Hinton Agar (MHA) and maintained in Mueller–Hinton Broth (MHB) at 37 °C for 24 hours, while fungal strains were maintained under similar conditions at 30 °C. The antimicrobial efficacy of the test compounds was assessed using the agar well diffusion method, in accordance with Clinical and Laboratory Standards Institute (CLSI) guidelines (2012).^40^ Briefly, the inoculum was uniformly spread over the agar surface, and wells of 6–8 mm diameter were aseptically punched into the agar. Each well was loaded with 20–100 µL of the test compound. The plates were then incubated at the respective optimal temperatures for 24 hours, after which the zones of inhibition were measured to evaluate antimicrobial activity.

### 3.5. *In vitro* cytotoxicity studies on normal and cancer cell lines

#### 3.5.1. Cytotoxicity assay

The cytotoxic potential of the synthesized compound 4k was evaluated using a panel of cell lines, including CHO-K1 (Chinese hamster ovary; normal), HEK-293 (human embryonic kidney; normal), SKBR3 (human breast cancer), HepG2 (human hepatoblastoma), and DU145 (human prostate cancer), following previously published protocols.^41^ All cell lines were obtained from the American Type Culture Collection (ATCC), USA and maintained in-house for ongoing drug screening programs at CSIR-Indian Indian Institute of Chemical Technology, Hyderabad, India. Initially, cells were seeded into 96-well plates and incubated overnight under standard culture conditions (37 °C, 5% CO_2_, humidified atmosphere). The following day, cells were treated with serial dilutions of compound 4k for 24 hours. After treatment, MTT reagent [3-(4,5-dimethylthiazol-2-yl)-2,5-diphenyltetrazolium bromide] was added to each well at a final concentration of 0.5 mg mL^−1^, and cells were incubated for 4 hours in the dark. Subsequently, 100 µL of DMSO : methanol (1 : 1, v/v) was added to dissolve the resulting formazan crystals. Cell viability was determined by measuring absorbance at 570 nm using a TECAN Infinite M200 Pro microplate reader. The IC_50_ values were calculated by linear regression analysis using data from three independent experiments.

### 3.6. Antioxidant assay

The antioxidant activity of 4k was evaluated using the 2,2-diphenyl-1-picrylhydrazyl (DPPH) radical scavenging assay, following our previously published method.^42^4k was tested at seven concentrations (10, 20, 40, 60, 80, 100, and 200 µM), each prepared in methanol. Rutin (100 µM) served as the reference standard. For each assay, 200 µL of the test solution was mixed with 2 mL of 0.1 mM DPPH solution in methanol, and the total volume was adjusted to 3 mL using methanol. The mixtures were incubated in the dark at room temperature for 20 minutes. After incubation, the absorbance was recorded at 517 nm using a UV-visible spectrophotometer. Methanol was used as the blank, and Rutin was processed under identical conditions for comparison.

The DPPH free radical scavenging activity (FRSA) of 4k was calculated using the following formula: FRSA= (*A*_c_ − *A*_s_/*A*_c_) × 100.

Where ‘*A*_c_’ is the absorbance of the control (DPPH solution without sample) and ‘*A*_s_’ is the absorbance of the sample. The experiment was performed in triplicate, and the average values were used to determine the antioxidant activity of 4k.

### 3.7. *In vitro* genotoxicity studies

#### 3.7.1. Chromosome aberration (CA) test

To assess the dose-dependent clastogenic potential of 4k, Chinese hamster ovary (CHO-K1) cells were treated with three sub-lethal concentrations (2.5, 5, and 7.5 µM), selected based on the IC_50_ concentration. The chromosomal aberration assay was performed following previously established protocols.^28,41^ Once the cells reached approximately 70% confluence, they were treated with 4k for 24 hours. Metaphase arrest was induced by adding colchicine (0.02%) to the culture medium for 45 minutes. Subsequently, the cells were treated with a hypotonic solution at 37 °C for 20 minutes, followed by centrifugation at 2000 rpm for 5 minutes. The resulting cell pellet was fixed using Carnoy's fixative (methanol : acetic acid, 3 : 1). Chromosome spreads were prepared using the flame-drying method and stained with 10% Giemsa solution. For each treatment group, at least 100 well-spread metaphase plates were analysed in triplicate and compared statistically with the vehicle control. Mitomycin-C (2.5 µM) and 1% DMSO were used as the positive and vehicle controls, respectively.

#### 3.7.2. Mitotic index (MI) test

The mitotic index (MI) was employed to evaluate changes in cell proliferation rates resulting from the cytotoxic effects of the test compound. MI was calculated as the percentage of cells undergoing mitosis (*i.e.*, prophase, metaphase, anaphase, and telophase) relative to the total number of cells observed. Slide preparation followed the same protocol as used for the chromosomal aberration assay. For each treatment group, a minimum of 2000 cells were randomly scored in triplicate to determine the mitotic index.

#### 3.7.3. Micronuclei (MN) test

The micronucleus (MN) assay was conducted to assess the clastogenic and aneugenic potential of the test compound. Micronuclei are small, extranuclear bodies formed from acentric chromosomal fragments or whole chromosomes that fail to segregate properly during anaphase. In this study, CHO-K1 cells were treated with three concentrations of 4k for 24 hours. After treatment, the cells were washed with PBS and incubated for an additional 24 hours in fresh DMEM containing cytochalasin B (3 µg mL^−1^) to block cytokinesis and facilitate the identification of binucleated cells. Cells were then harvested and incubated in 1% sodium citrate buffer at 37 °C for 8–10 minutes to induce hypotonic swelling. Following centrifugation, the pellet was resuspended in fresh hypotonic solution. Thin cell smears were prepared on clean glass slides, air-dried, fixed with methanol, and stained with Giemsa. For each concentration, a minimum of 2000 cells were scored in triplicate to determine the frequency of micronucleated cells.

#### 3.7.4. Flow cytometric analysis of cell cycle modulation

Human cancer cell lines HepG2 and DU145 were seeded in 60 mm culture dishes and incubated at 37 °C in a humidified incubator with 5% CO_2_ for 24 hours. Following incubation, cells were treated with the test compound 4k at its respective IC_50_ concentrations (2.27 µM for HepG2 and 5.0 µM for DU145). In parallel, normal CHO-K1 cells were treated with a sublethal concentration of 7.5 µM 4k under identical conditions. After 24 hours of treatment, the cells were harvested, fixed in 70% ethanol, and stored at −20 °C for 24 hours. Fixed cells were then stained in 500 µL of staining buffer containing 10 µL of propidium iodide (2.5 mg mL^−1^), 1 µL of RNase A, and 0.5% Triton X-100, and incubated in the dark at room temperature for 40 minutes. Subsequently, the stained cells were centrifuged, resuspended in 500 µL PBS, and analyzed for cell cycle distribution using flow cytometry (FACS).

## 4 Conclusion

This overall study suggests that 4k exhibits good antioxidant and selective anticancer activities *in vitro*. It reduces erythrocyte osmotic fragility, protects normal cells from oxidative damage, and found to be non-genotoxic in nature toward normal cells, indicating a favorable preliminary safety profile. These findings also support the potential of 4k in oxidative stress-related conditions, including diabetes and hepatic cancer. However, further studies are required to evaluate its *in vivo* efficacy, molecular mechanisms, and formulation aspects before considering possible clinical applications.

## Author contributions

KV: performed synthesis. JM: formal analysis of anti-cancer activity, data curation. RB: formal analysis of anti-microbial activity, data curation. ANK: conceptualization, performed synthesis, writing first draft of manuscript. AF: formal analysis of anti-diabetic activity, data curation. NM: formal analysis of erythrocyte osmotic fragility, data curation. KK: writing-original draft, validation, supervision, resources, project administration, methodology, formal analysis, conceptualization. KVNSS: writing-original draft, validation, supervision, methodology, conceptualization. SM: supervision, resources, formal analysis of anti-cancer and anti-microbial activity, data curation, conceptualization. SL: performed erythrocyte osmotic fragility analysis, data curation. AZ: supervision, resources, formal analysis of anti-diabetic activity, data curation, conceptualization. BB: performed spectral analysis.

## Conflicts of interest

Authors declare no conflict of interest.

## Ethical statement

All experiments were conducted in accordance with national and institutional ethical guidelines and were approved by the Ethics Committee of CSIR–Central Institute of Medicinal and Aromatic Plants (CSIR-CIMAP) under the approved protocol number CIMAP/IHE/2022/01. Written informed consent was obtained from all human participants involved in the study.

## Supplementary Material

RA-016-D6RA03816F-s001

## Data Availability

The data that support the findings of this study are available upon request. Supplementary information (SI): experimental data and spectroscopy data of all the synthesized compounds such as IR, NMR and Mass. See DOI: https://doi.org/10.1039/d6ra03816f.
