# Supplementary material for: Design, synthesis, and biological evaluation of vanillin–piperidone hybrids with potent anticancer activity and a favourable genotoxic profile
Source: RSC Adv. 2026 Jul 3;16(35):36019–32. doi: 10.1039/d6ra03816f (PMC13330124; doi:10.1039/d6ra03816f)
Supplement: RA-016-D6RA03816F-s001 [file RA-016-D6RA03816F-s001.pdf]

Supplementary Information (SI) for RSC Advances.
This journal is © The Royal Society of Chemistry 2026

SUPPORTING DATA

Design, Synthesis, and Biological Evaluation of Vanillin-Piperidone Hybrids with Potent Anticancer Activity and a Favourable Genotoxic Profile

M. Krishna Vamsi[a, #], Jhansi Mamilla[b,$,#], Ramya Bandari[b], A. Niranjan Kumar[a], Afra Fatima[b], Nidhi Maurya[c], J Kotesh Kumar[a, $, *], K.V.N.S. Srinivas[a], Sunil Misra[b, $], Suaib Luqman[c], Amtul Zehra[b], B. Balakishan,[d]

[a]Phytochemistry Division, CSIR-Central Institute of Medicinal and Aromatic Plants, Research Centre, Boduppal, Hyderabad-500 092, India. [koteshkumarj@cimap.res.in; kvn.satyasrinivas@cimap.res.in];
[b]Department of Applied Biology, CSIR-Indian Institute of Chemical Technology, Hyderabad-500007, India;
[c]Bio-Prospection and Product Development, CSIR-Central Institute of Medicinal and Aromatic Plants, Lucknow-226015, India;
[d]NMR Division, CSIR-Central Institute of Medicinal and Aromatic Plants, Lucknow-226015, India;
[$]Academy of Scientific and Innovative Research (AcSIR), Ghaziabad 201002, India.
# *equal contribution* M. Krishna Vamsi and Jhansi Mamilla

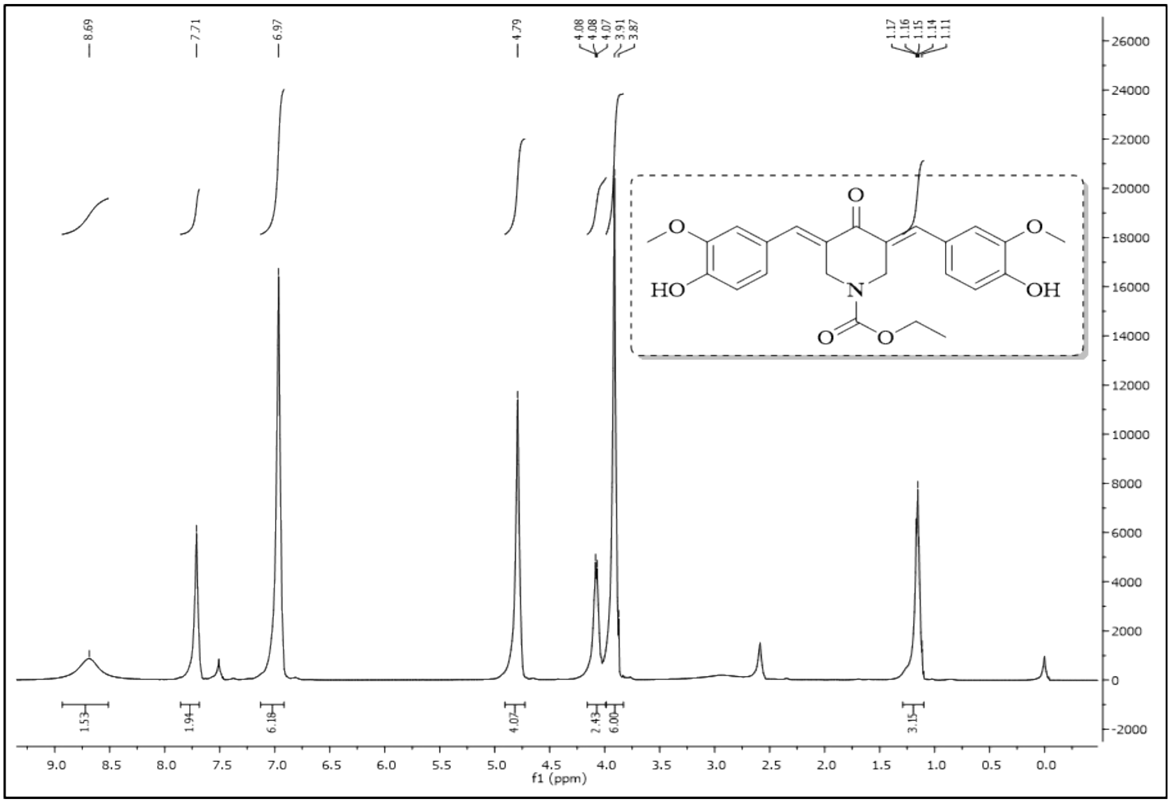

Figure S1: ^{1}H NMR Spectrum of compound **3**. [500 MHz, Solvent-$CDCl_3$]

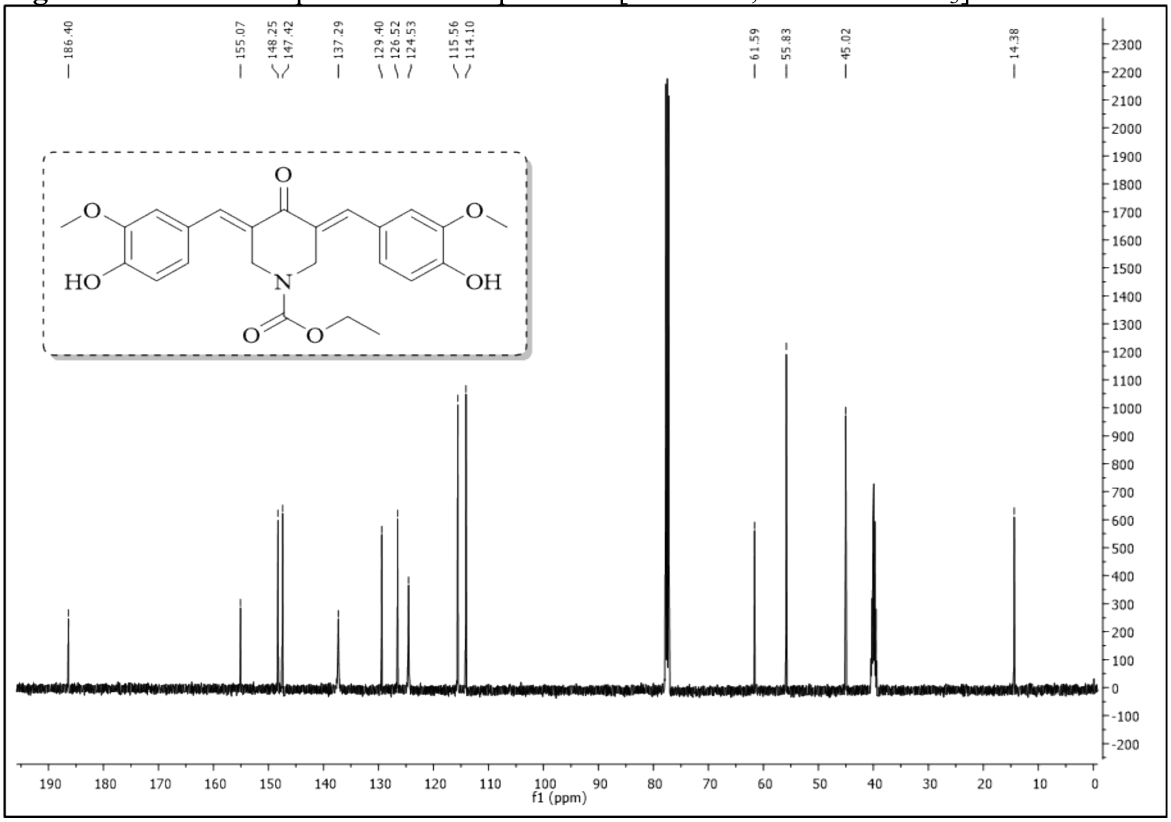

Figure S2: ^{13}C NMR Spectrum of compound **3**. [125 MHz, Solvent-$CDCl_3$]

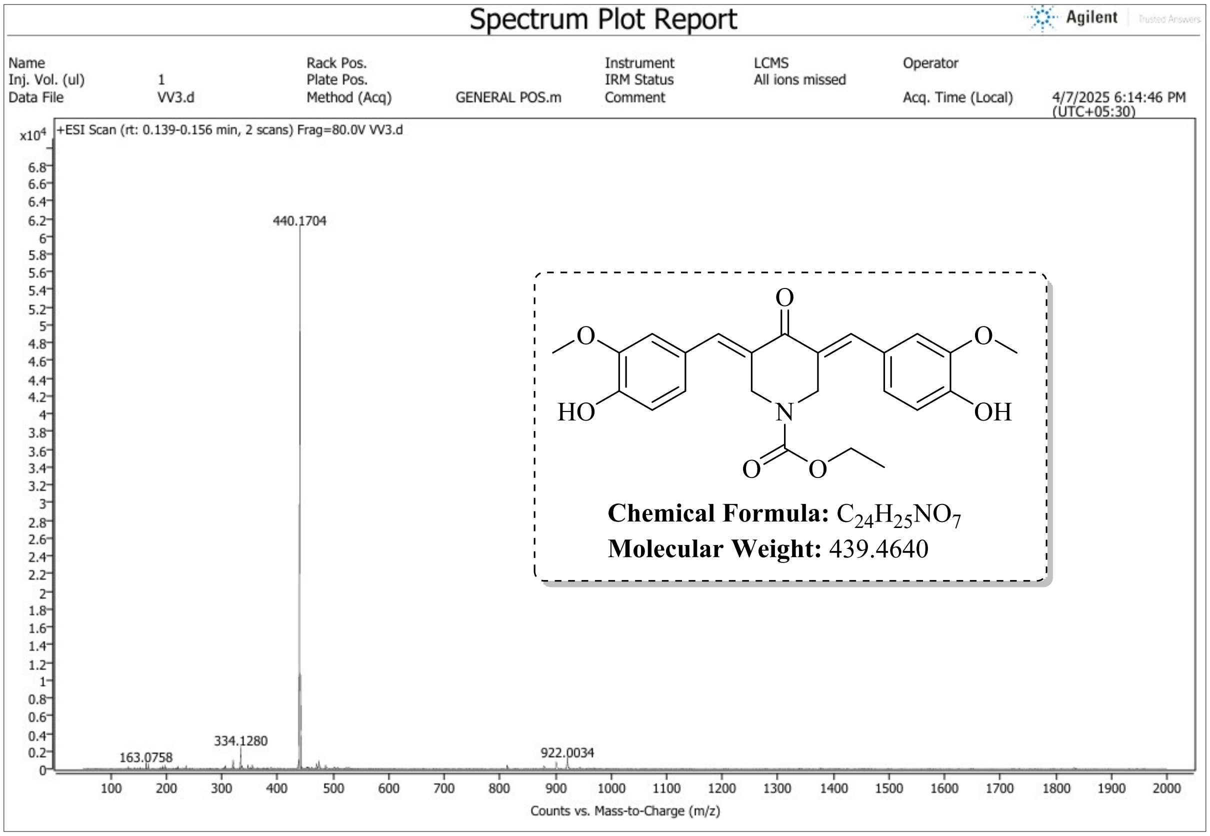

Figure S3: HR-MS Spectrum of compound **3**

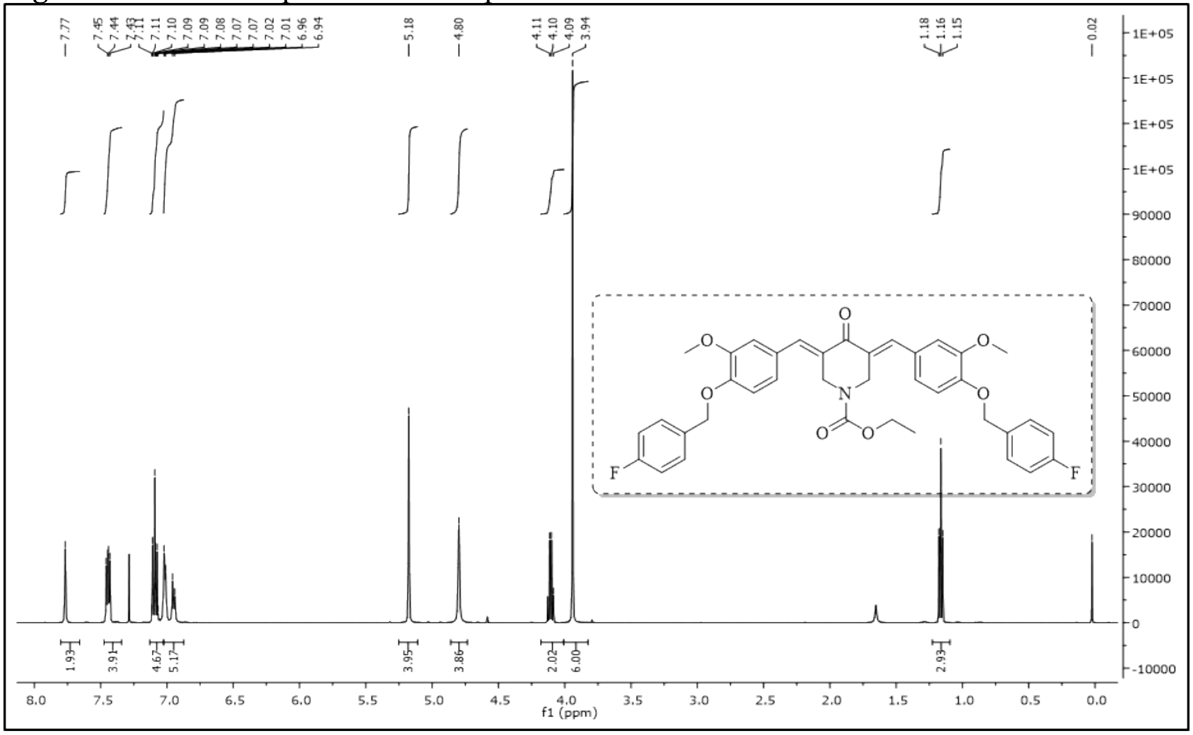

Figure S4: ^{1}H NMR Spectrum of compound **4a**. [500 MHz, Solvent-$CDCl_3$]

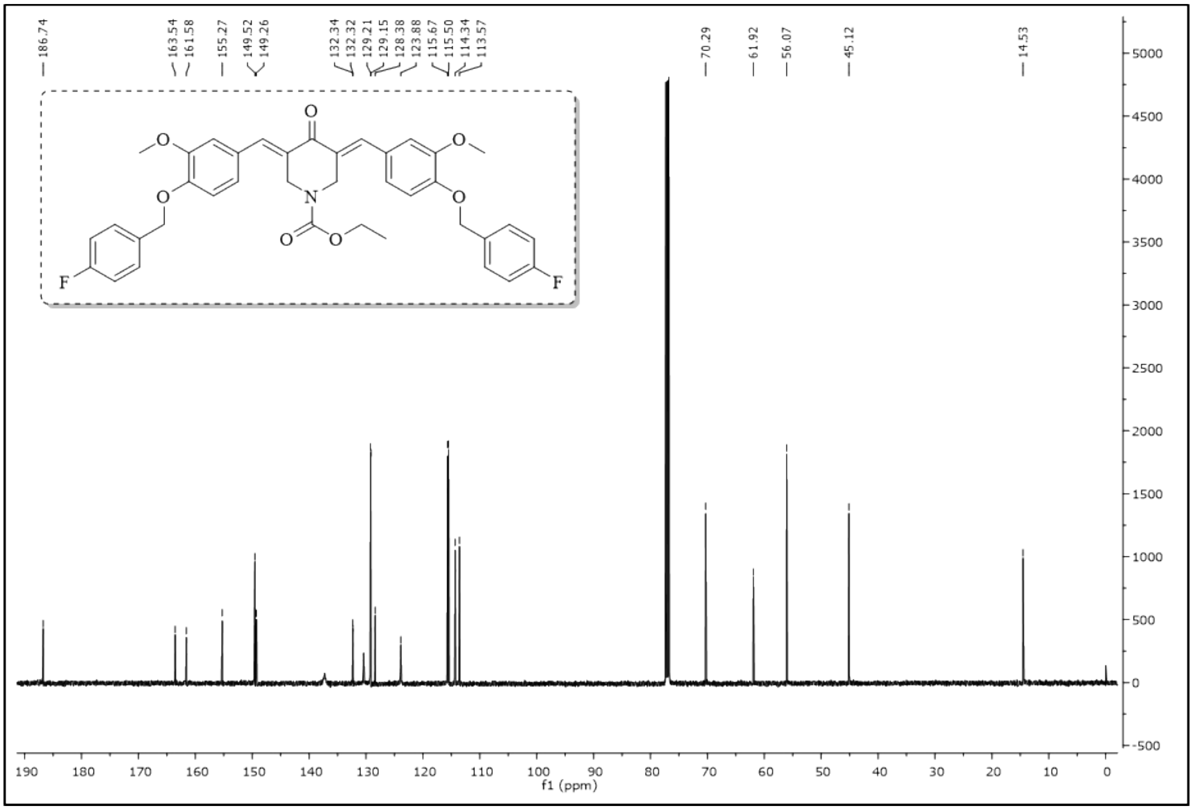

Figure S5: ^{13}C NMR Spectrum of compound **4a**. [125 MHz, Solvent-$CDCl_3$]

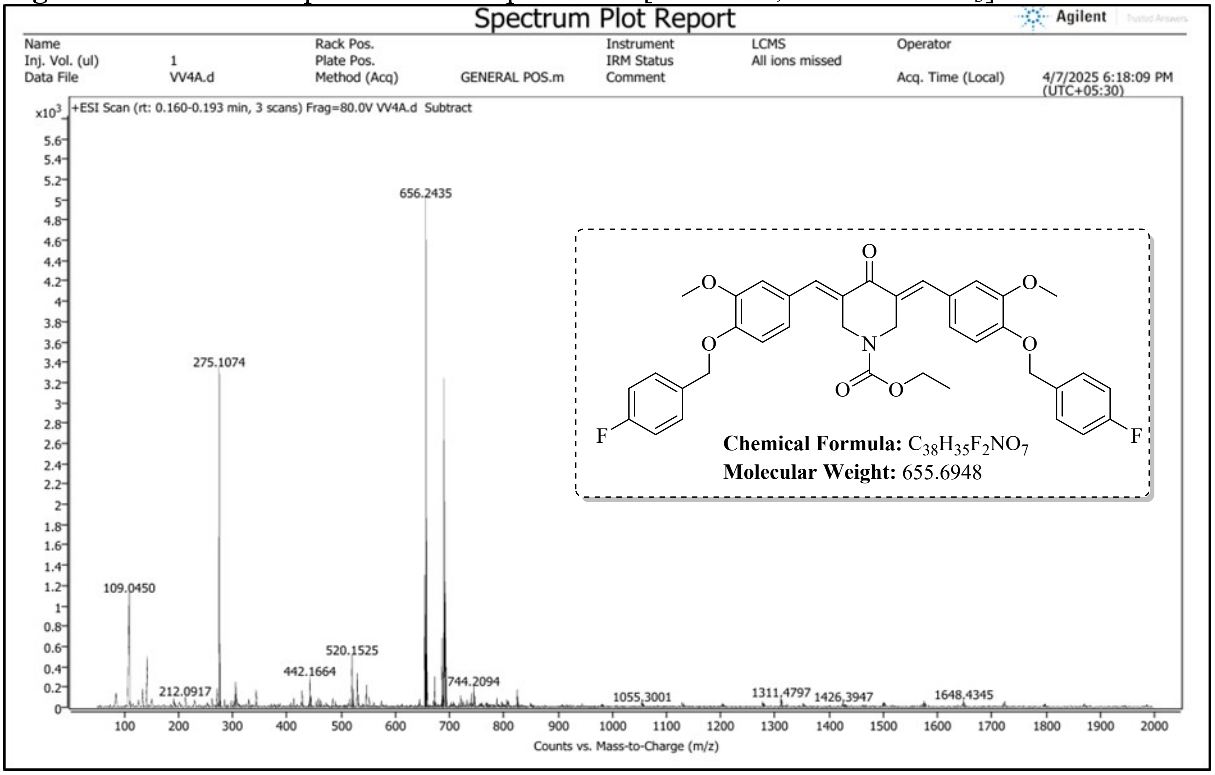

Figure S6: HR-MS Spectrum of compound **4a**

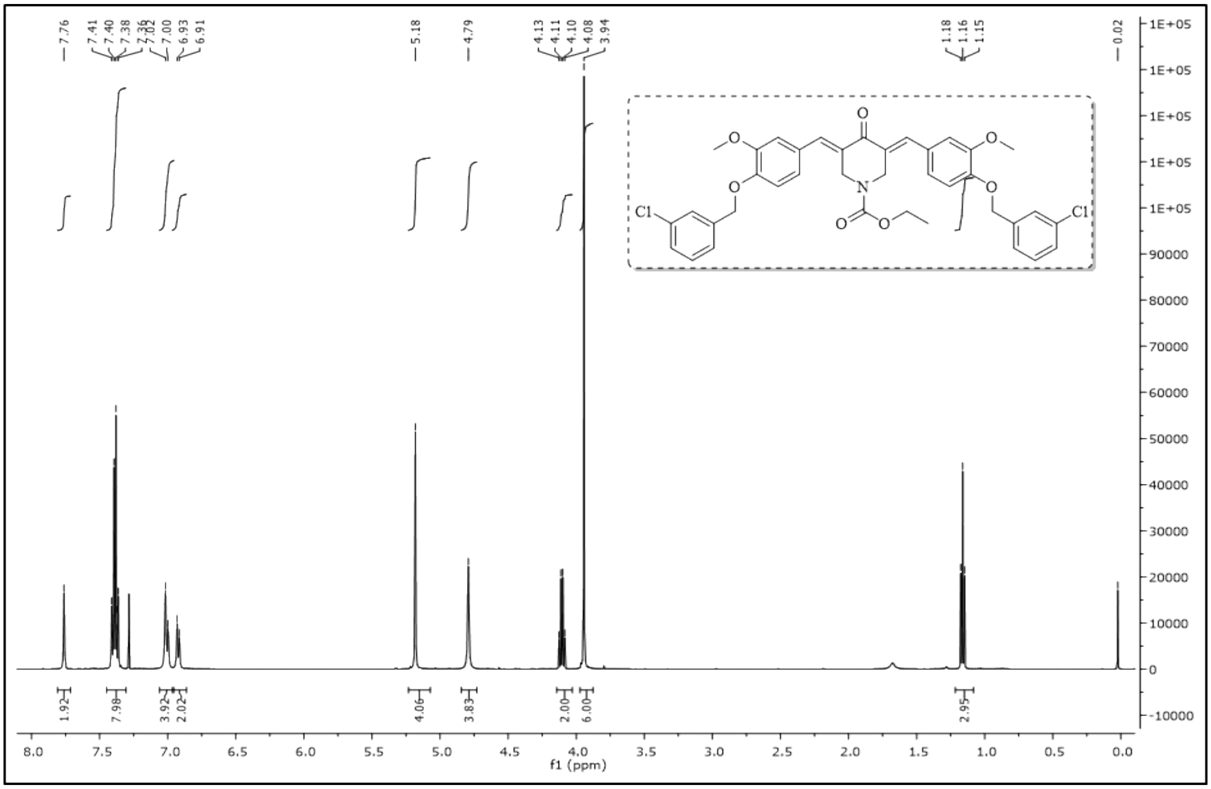

Figure S7: ^{1}H NMR Spectrum of compound **4b** [500 MHz, Solvent-$CDCl_3$]

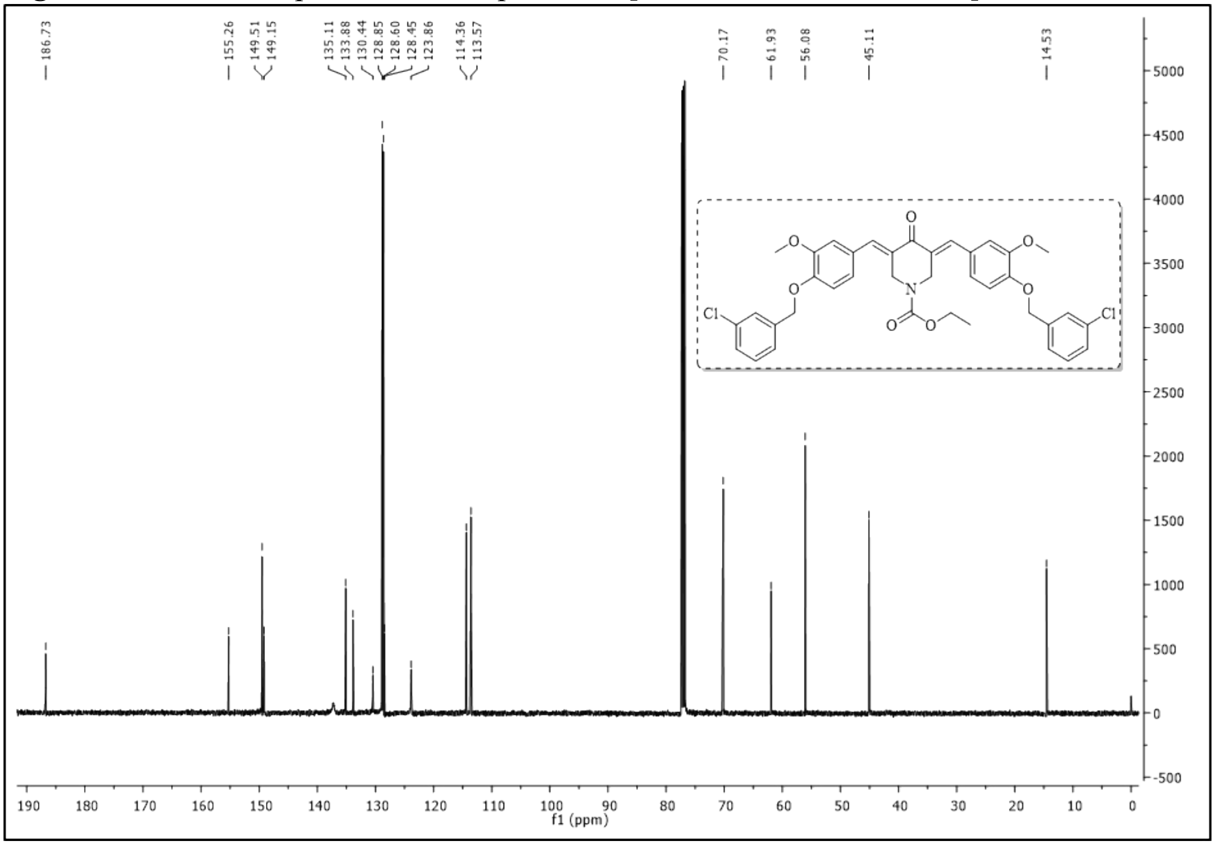

Figure S8: ^{13}C NMR Spectrum of compound **4b**. [125 MHz, Solvent-$CDCl_3$]

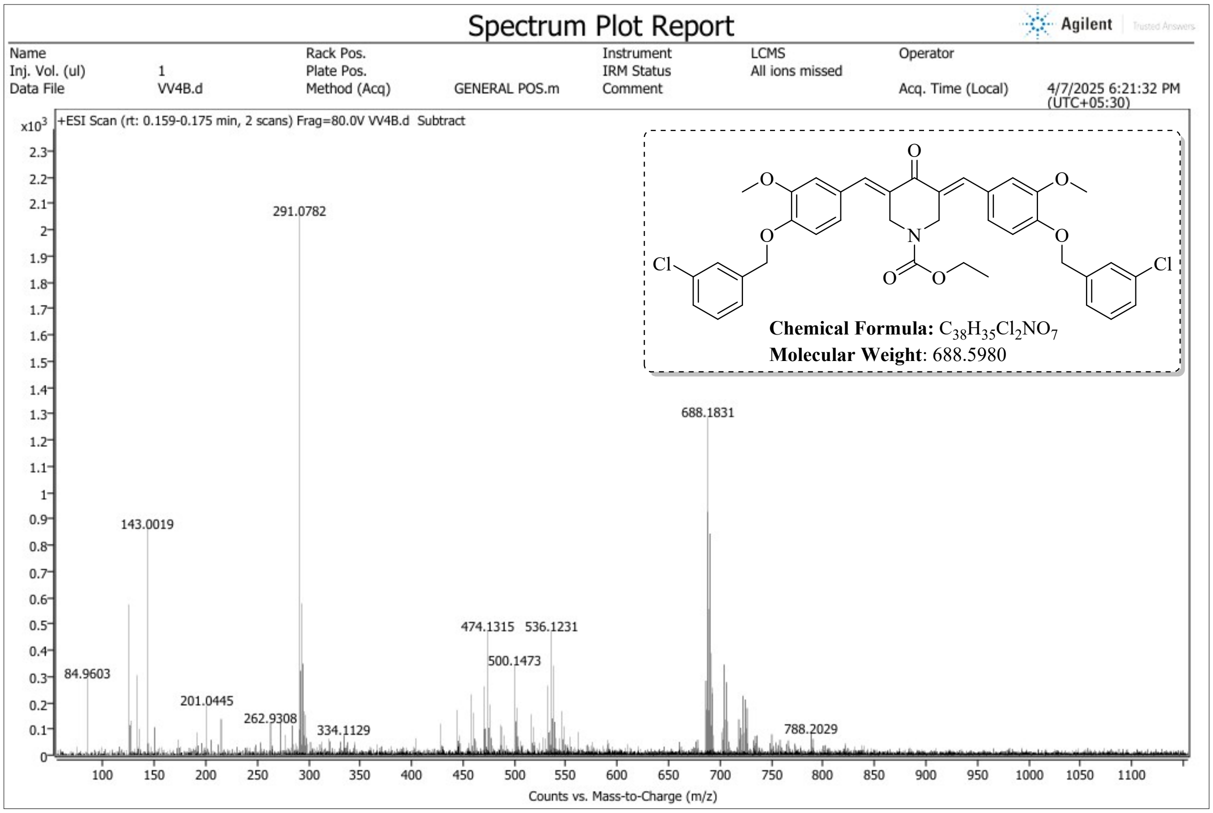

Figure S9: HR-MS Spectrum of compound **4b**

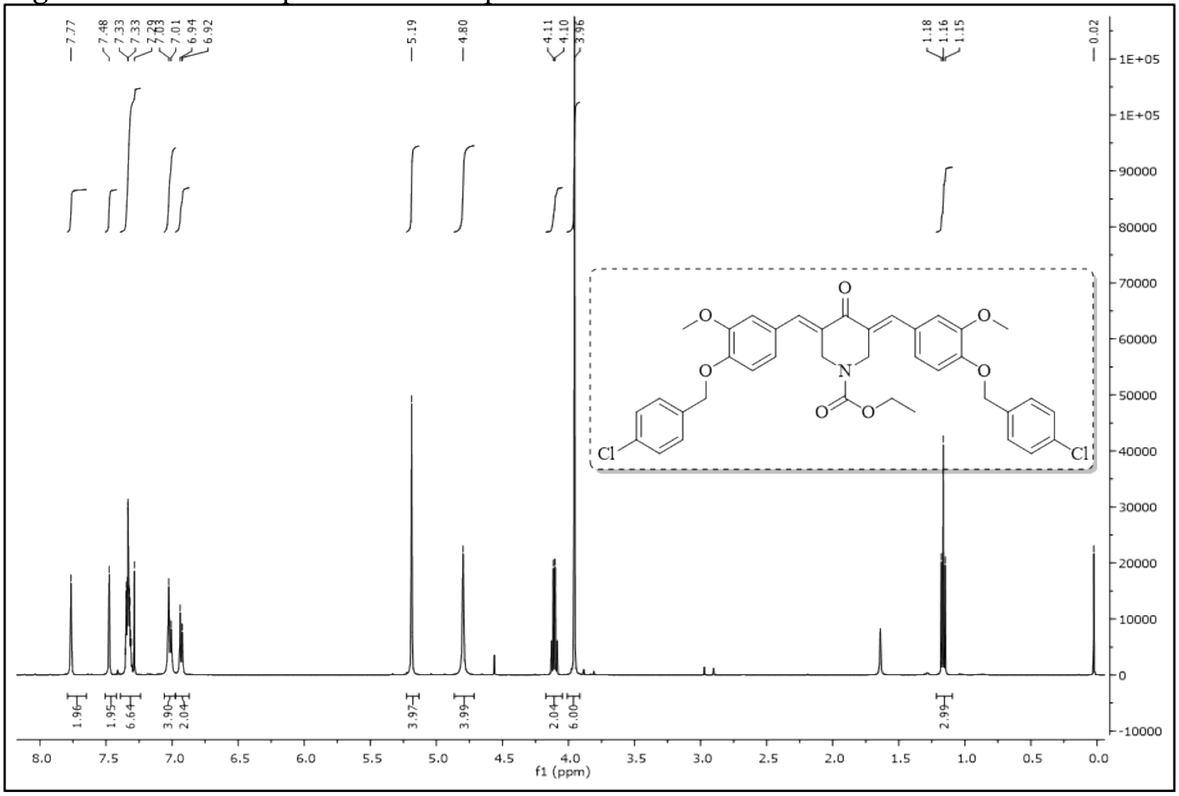

Figure S10: ^{1}H NMR Spectrum of compound **4c.** [500 MHz, Solvent-$CDCl_3$]

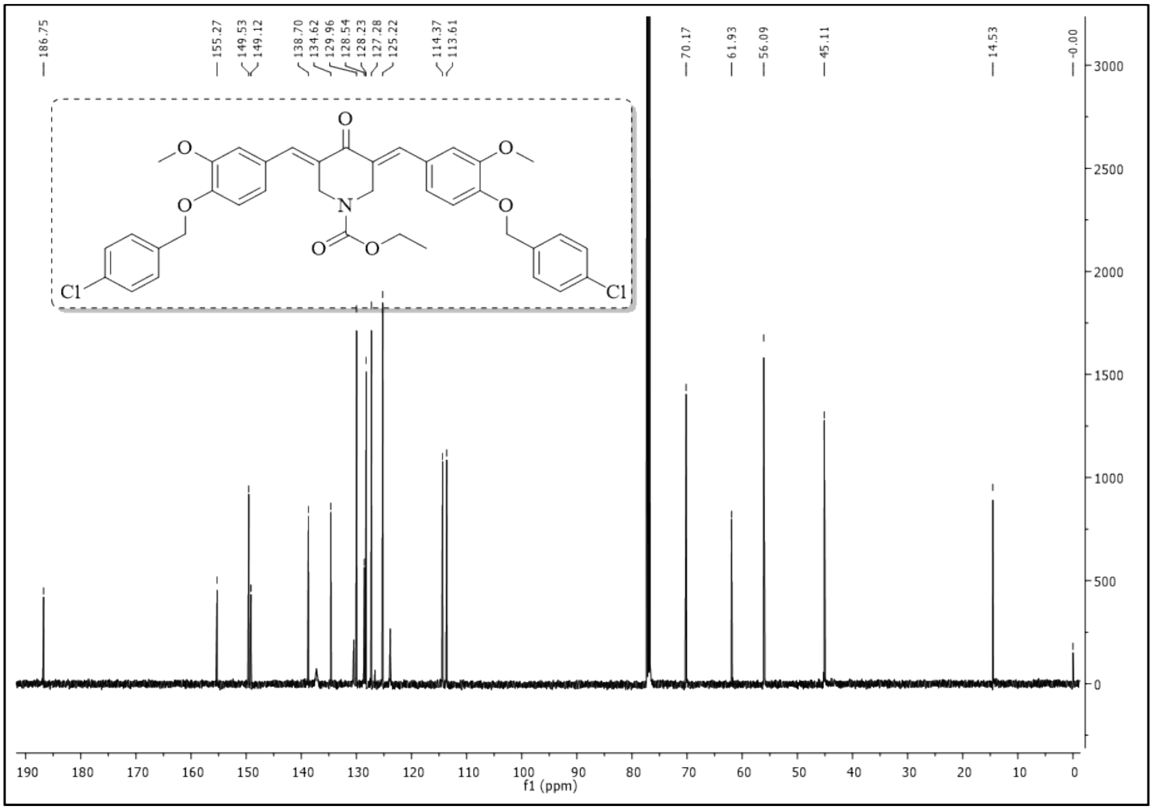

Figure S11: ^{13}C NMR Spectrum of compound **4c**. [125 MHz, Solvent-$CDCl_3$]

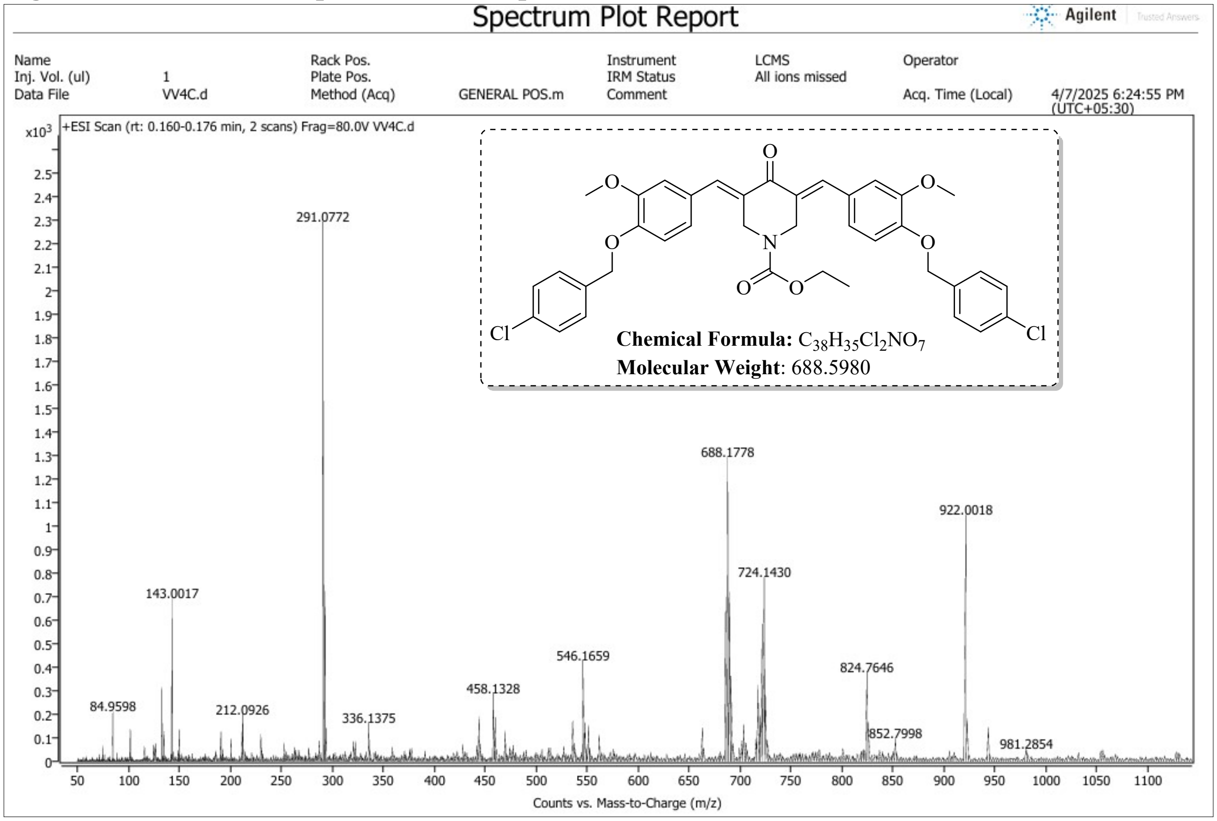

Figure S12: HR-MS Spectrum of compound **4c**

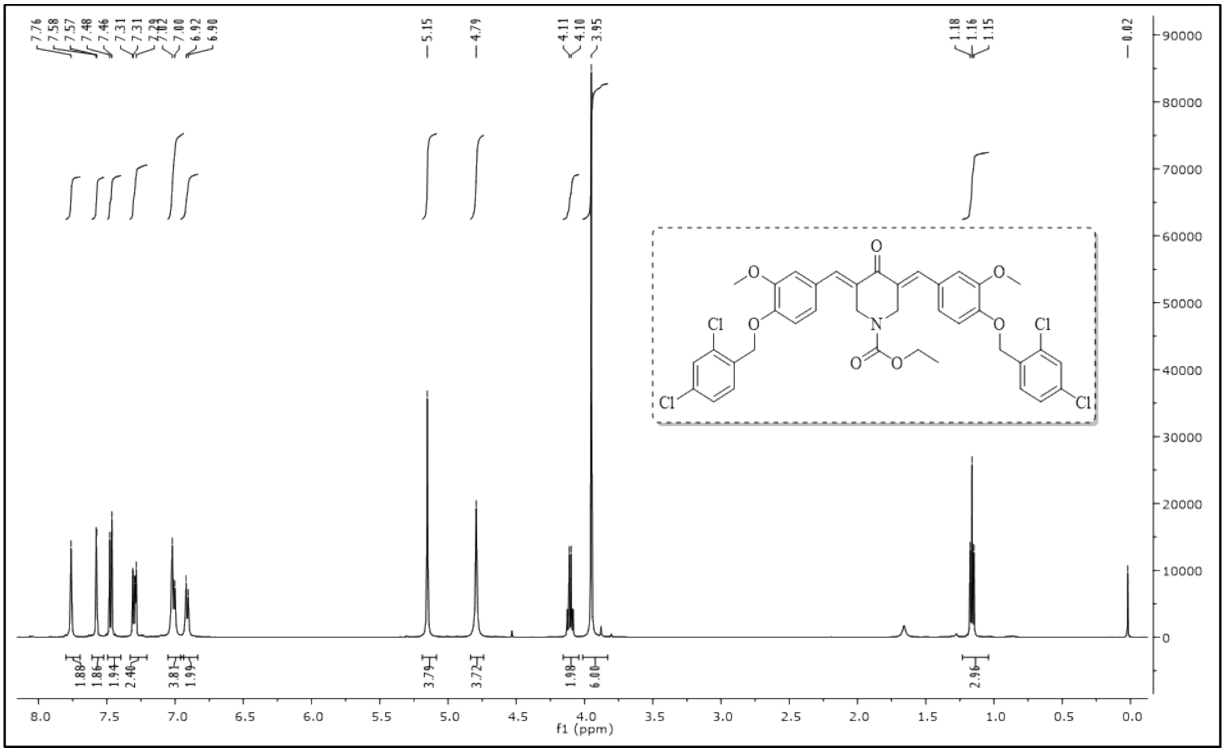

Figure S13: ^{1}H NMR Spectrum of compound **4d** [500 MHz, Solvent-$CDCl_3$]

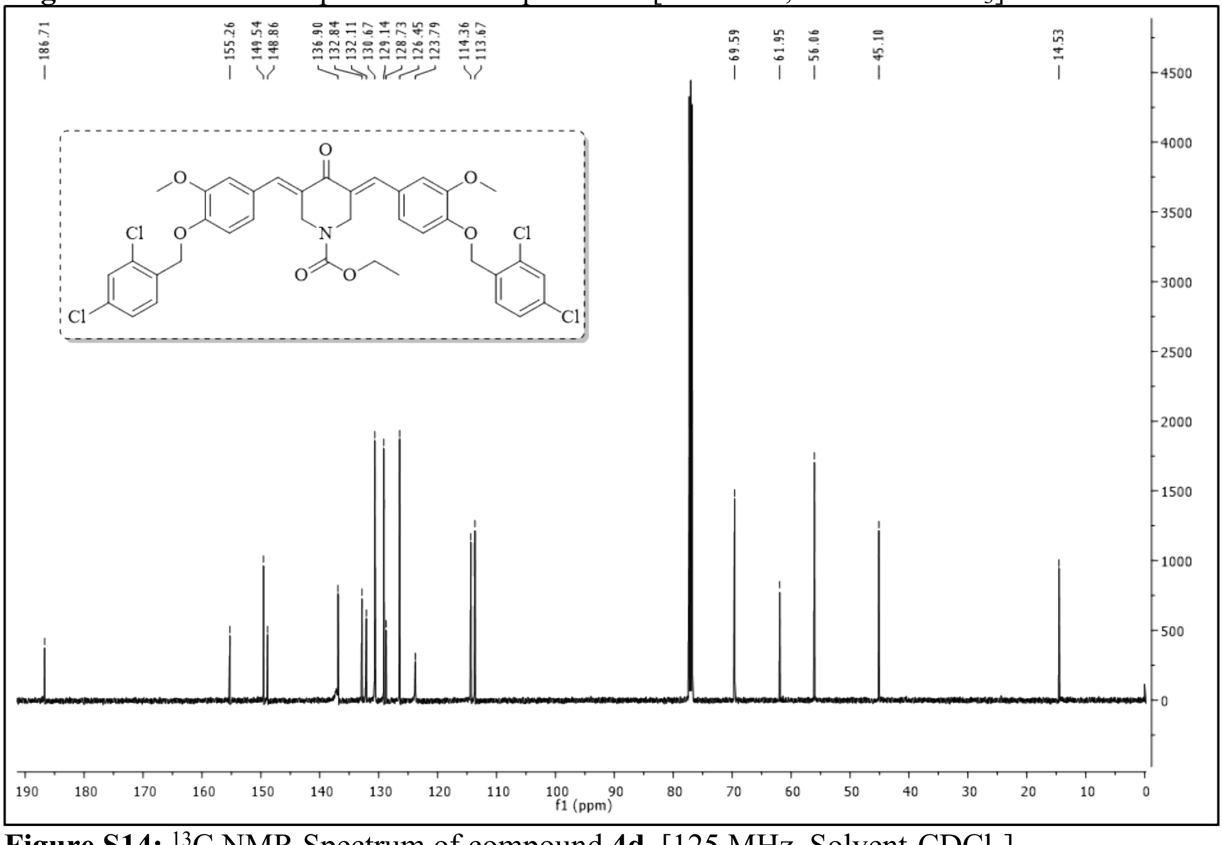

Figure S14: ^{13}C NMR Spectrum of compound **4d**. [125 MHz, Solvent-$CDCl_3$]

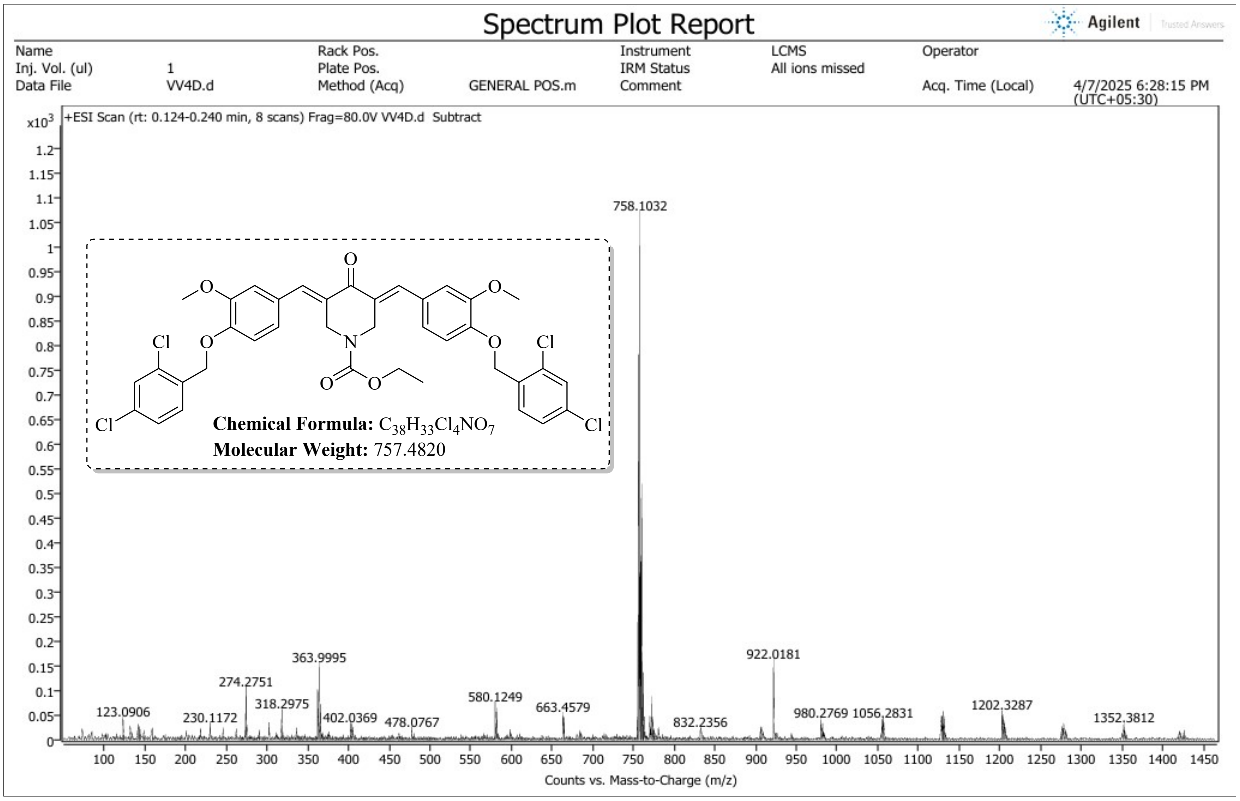

Figure S15: HR-MS Spectrum of compound **4d**

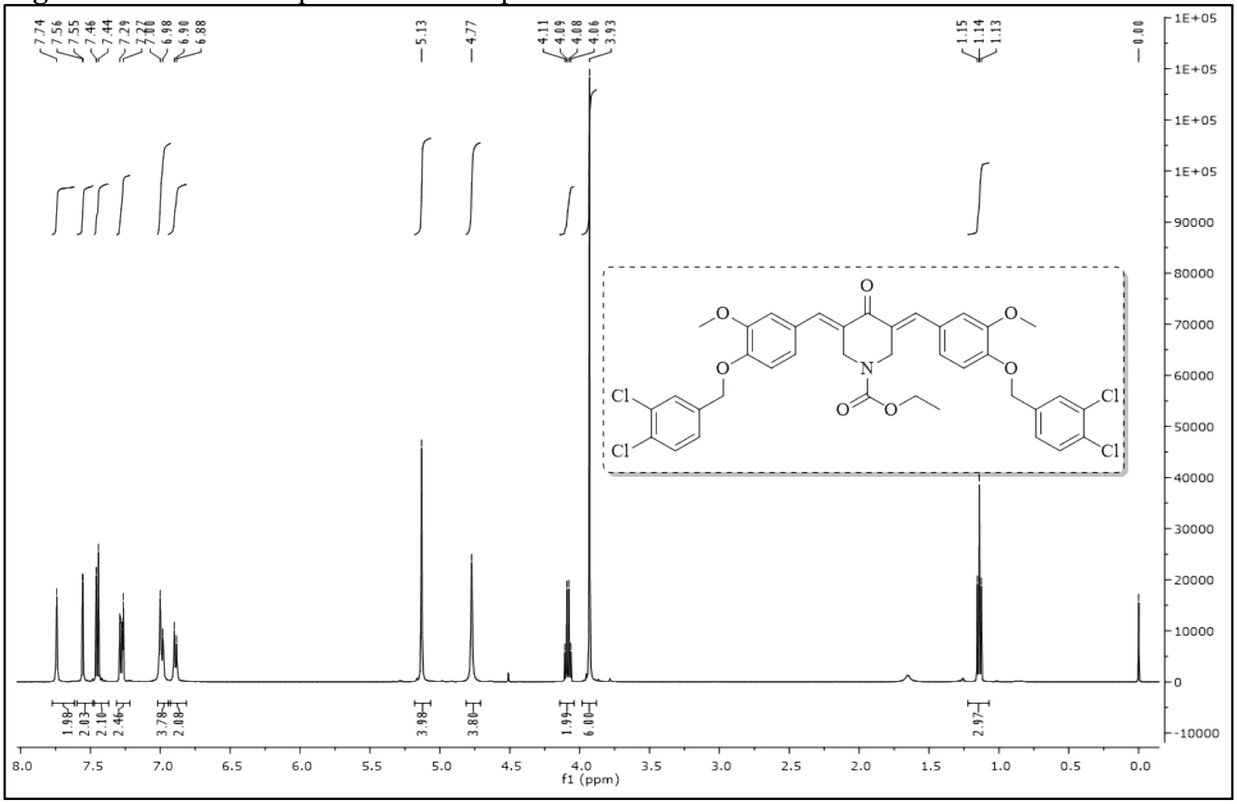

Figure S16: ^{1}H NMR Spectrum of compound **4e** [500 MHz, Solvent-$CDCl_3$]

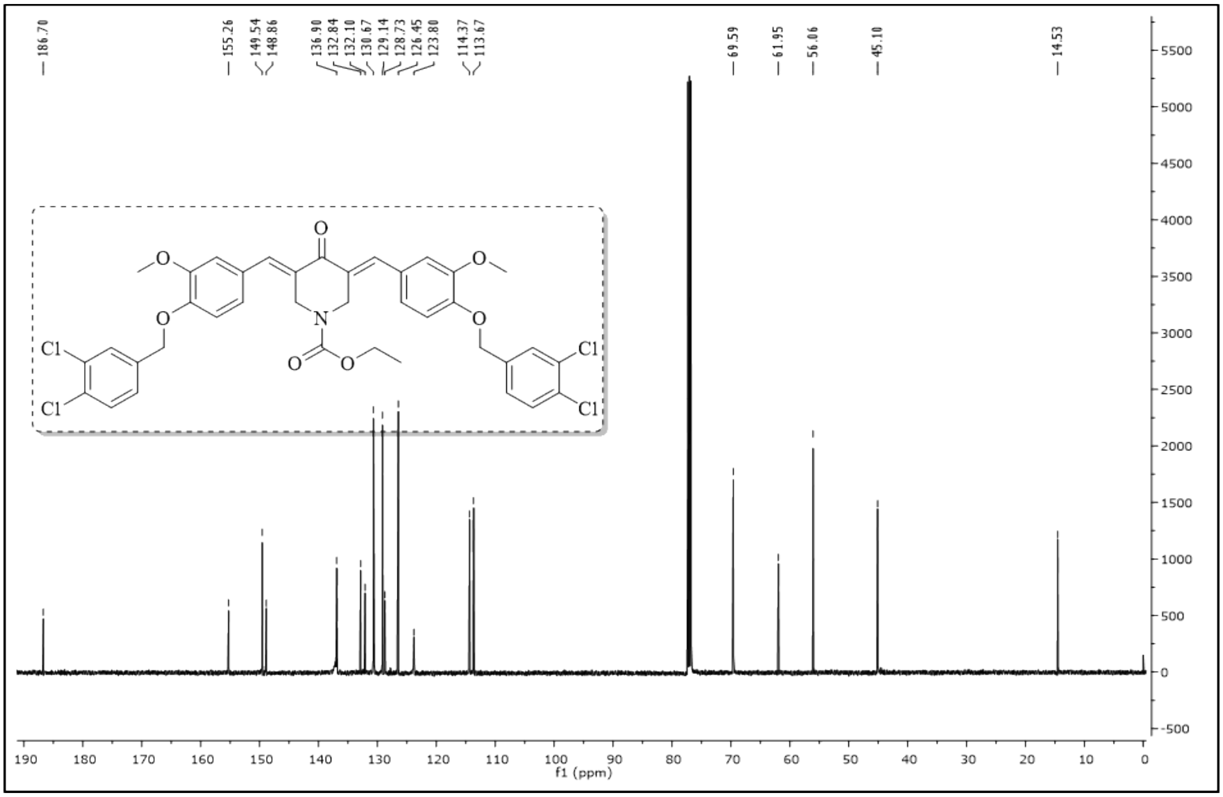

Figure S17: ^{13}C NMR Spectrum of compound **4e**. [125 MHz, Solvent-$CDCl_3$]

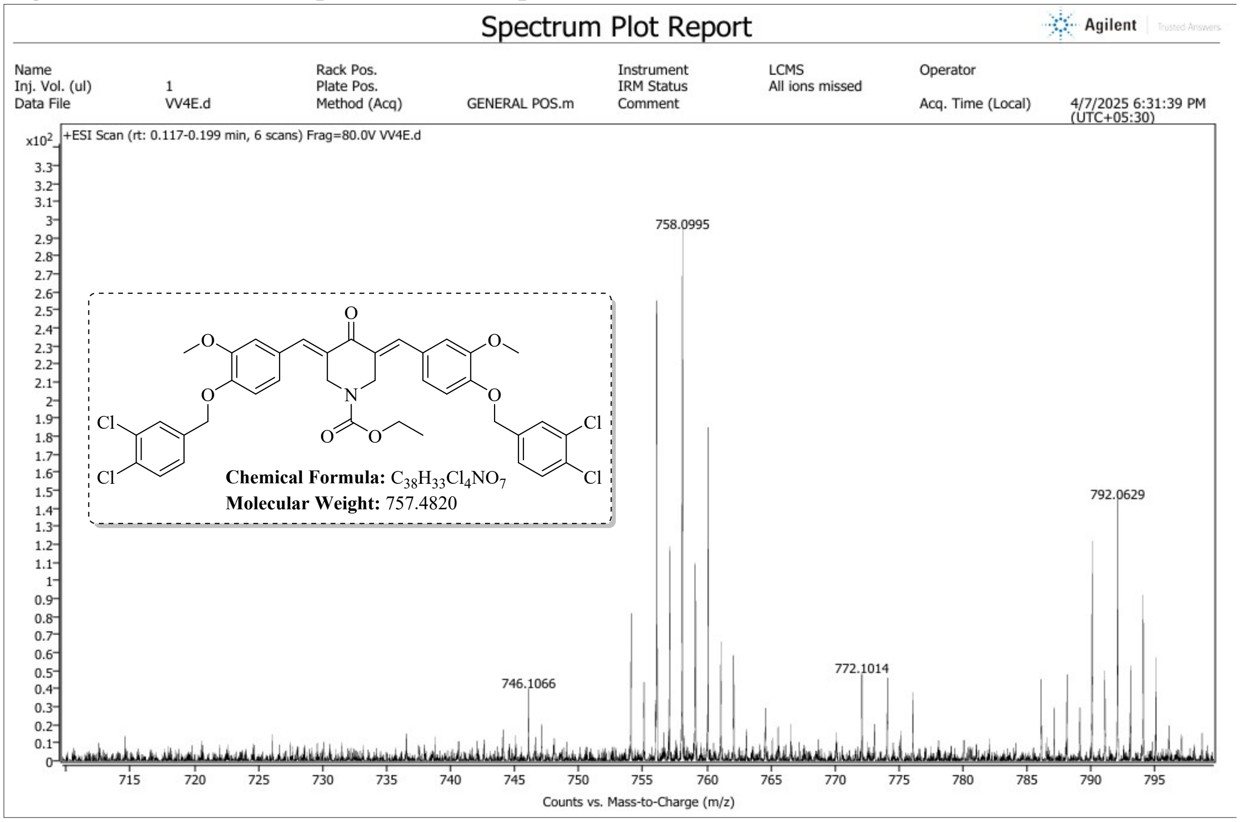

Figure S18: HR-MS Spectrum of compound **4e**

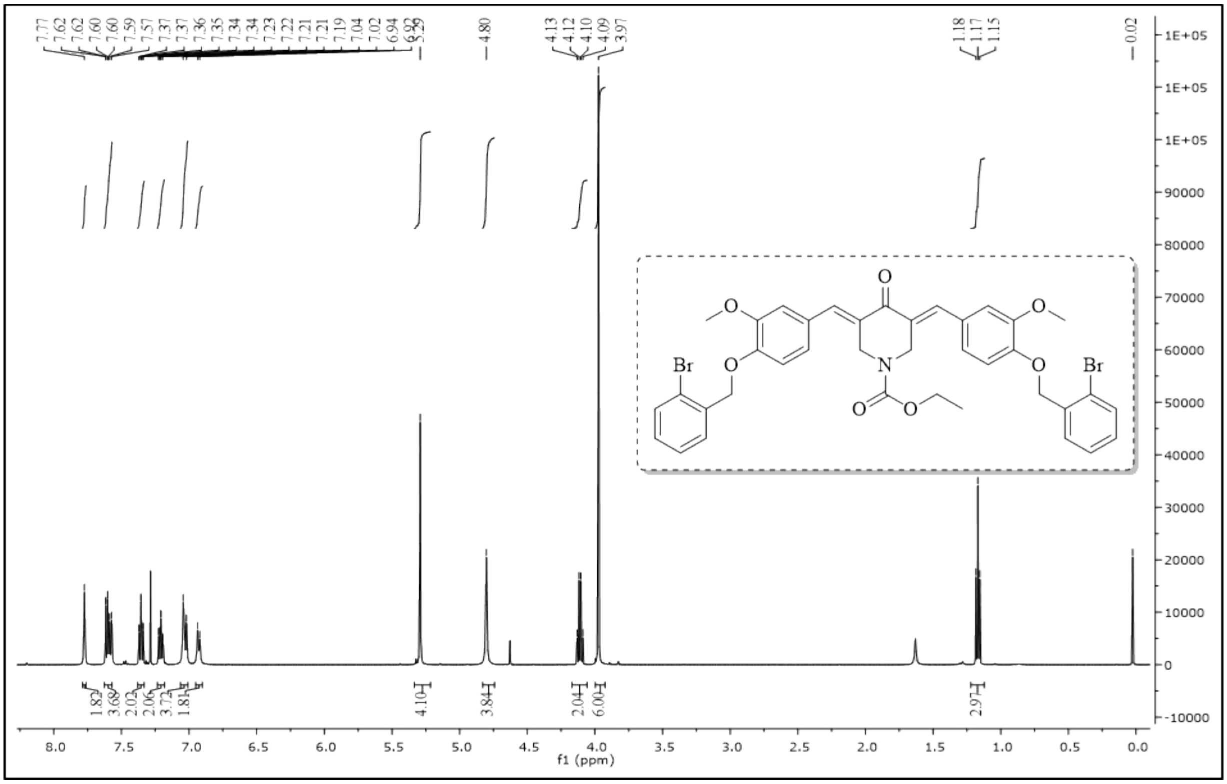

Figure S19: ^{1}H NMR Spectrum of compound **4f** [500 MHz, Solvent-$CDCl_3$]

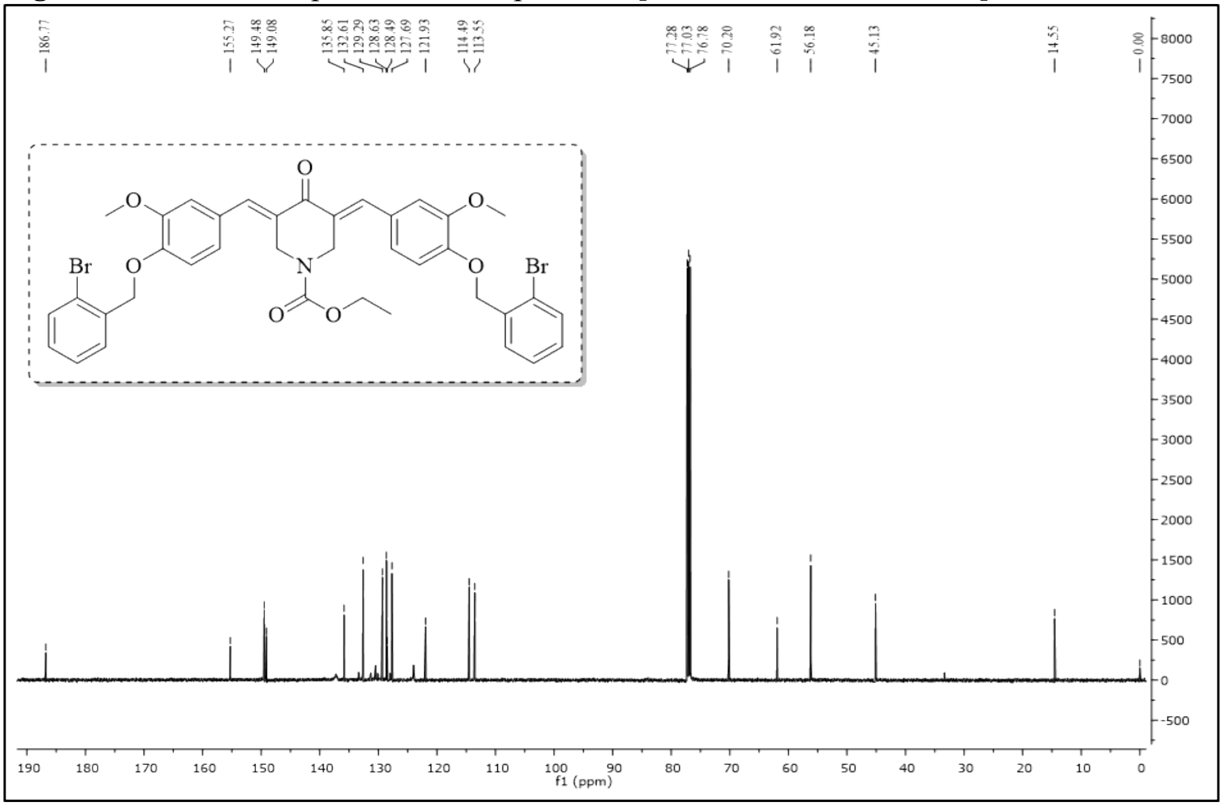

Figure S20: ^{13}C NMR Spectrum of compound **4f**. [125 MHz, Solvent-$CDCl_3$]

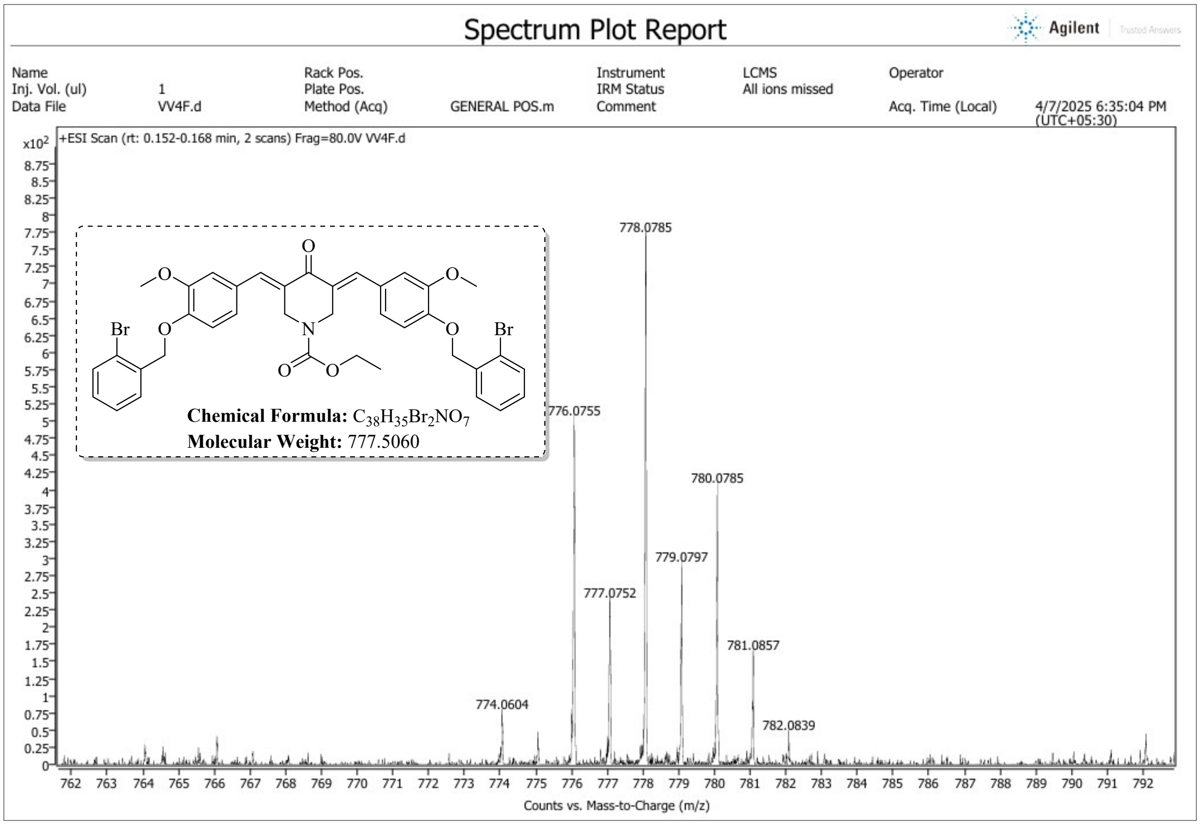

Figure S21: HR-MS Spectrum of compound **4f**

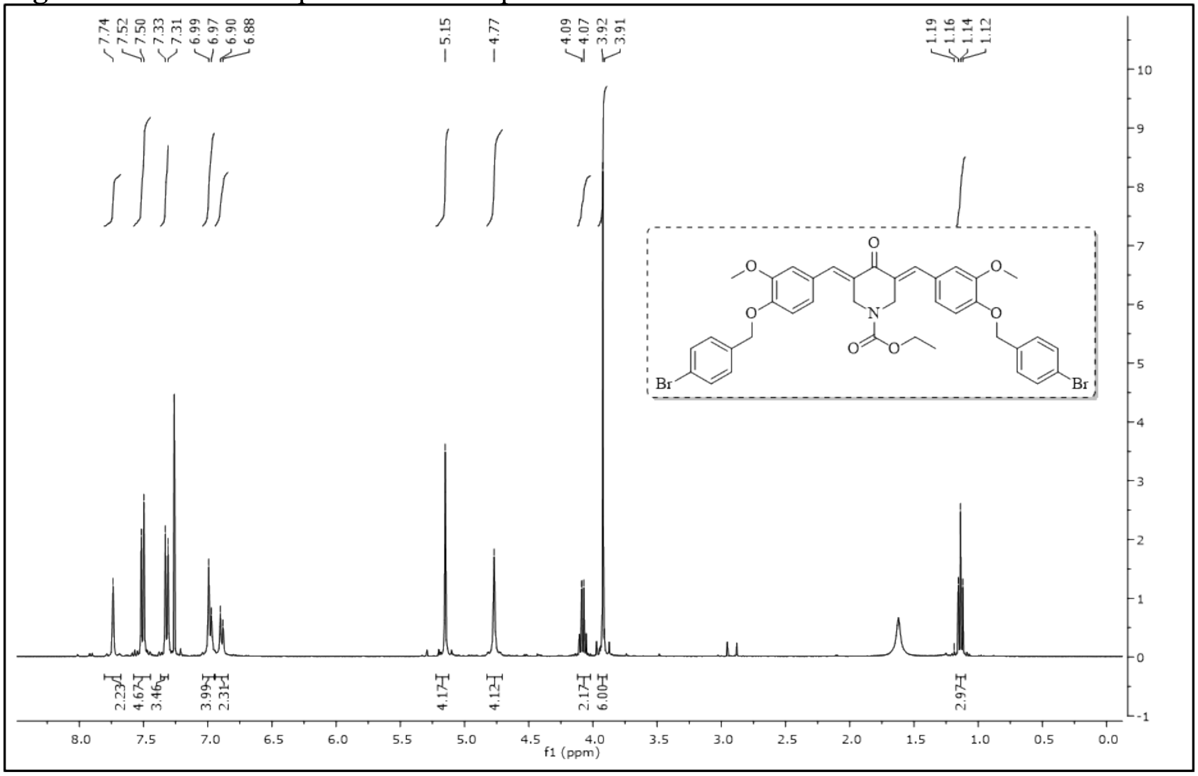

Figure S22: ^{1}H NMR Spectrum of compound **4g** [500 MHz, Solvent-$CDCl_3$]

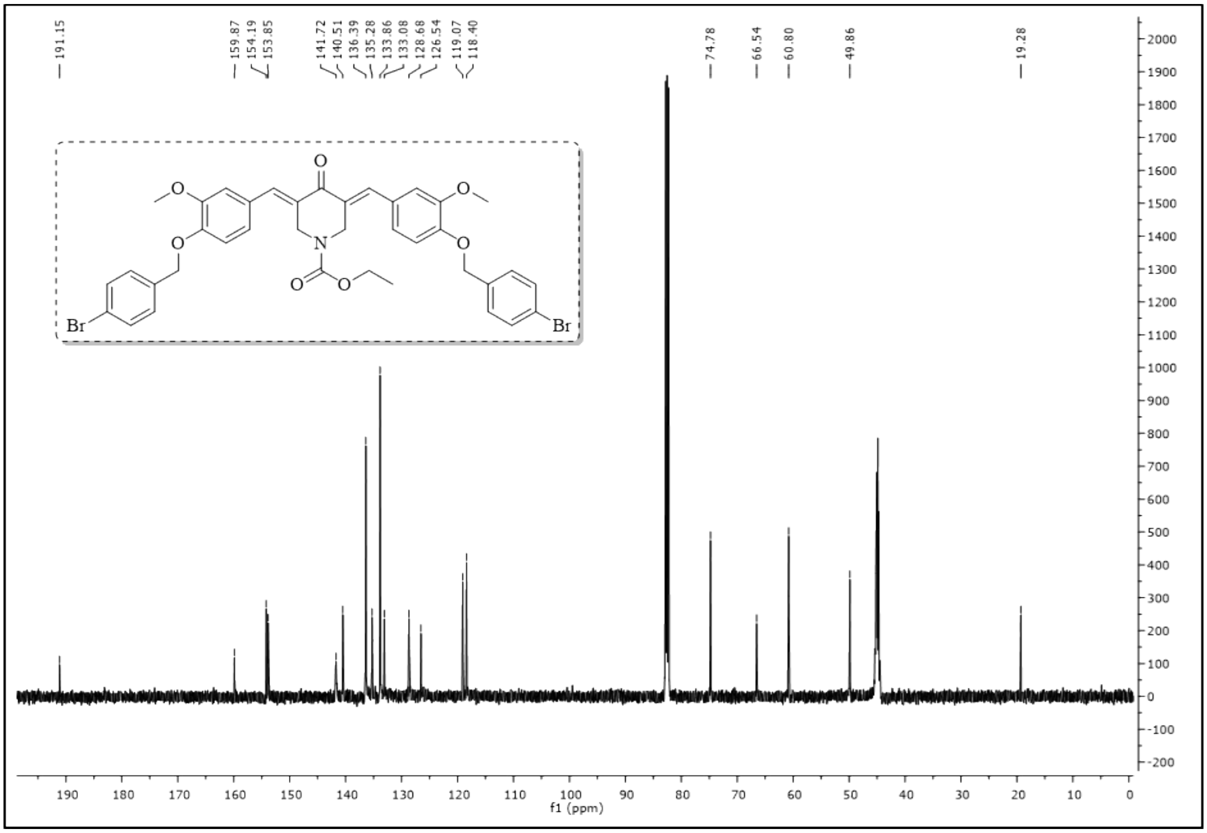

Figure S23: ^{13}C NMR Spectrum of compound **4g**. [125 MHz, Solvent-$CDCl_3$]

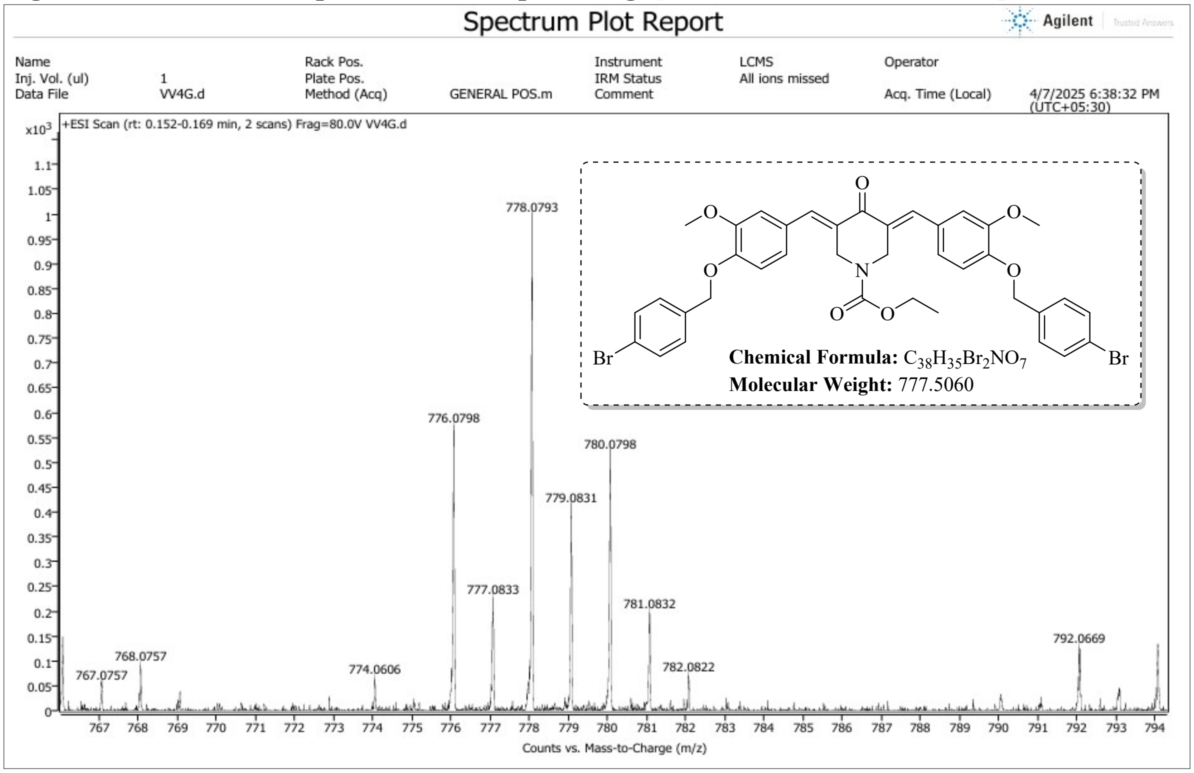

Figure S24: HR-MS Spectrum of compound **4g**

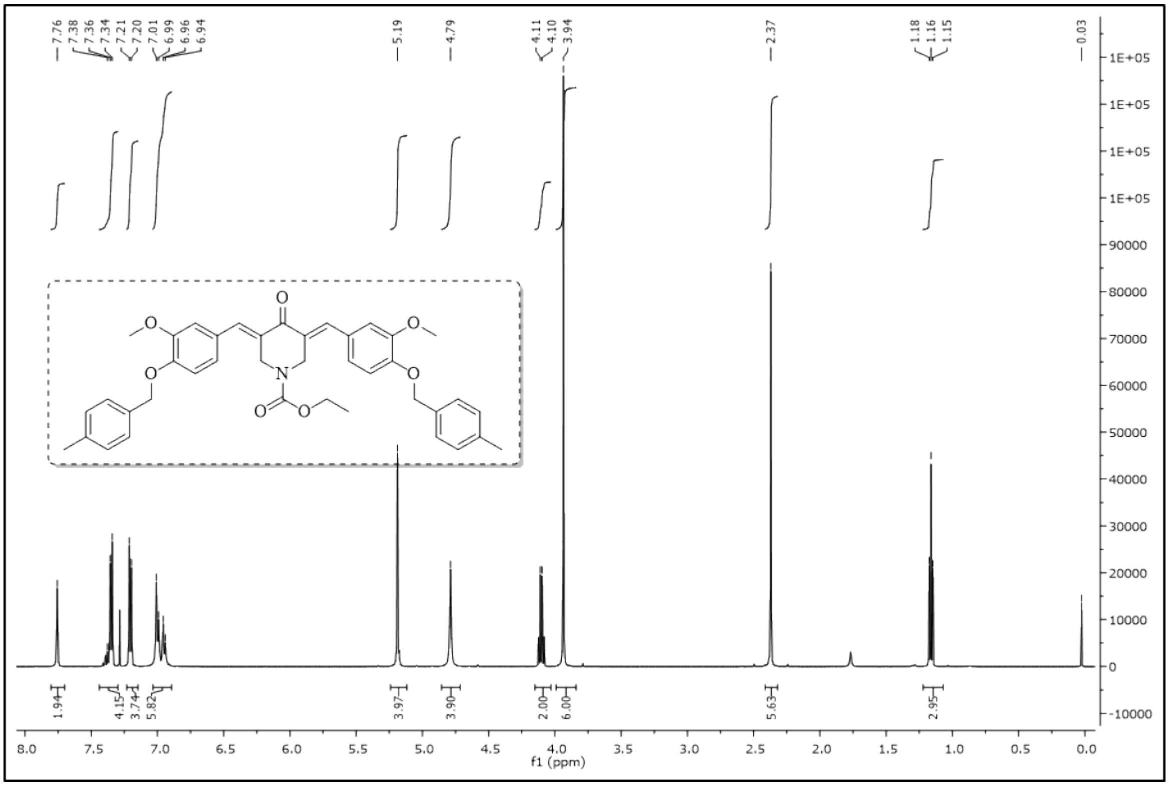

Figure S25: ^{1}H NMR Spectrum of compound **4h** [500 MHz, Solvent-$CDCl_3$]

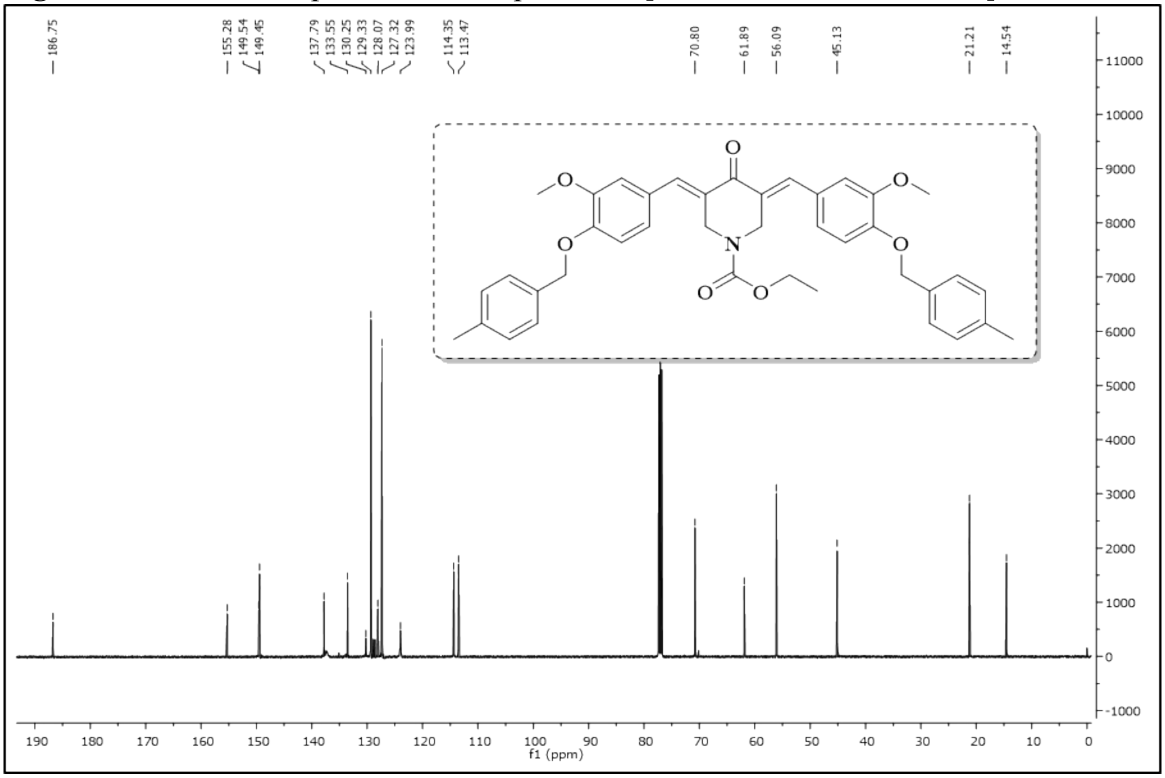

Figure S26: ^{13}C NMR Spectrum of compound **4h**. [125 MHz, Solvent-$CDCl_3$]

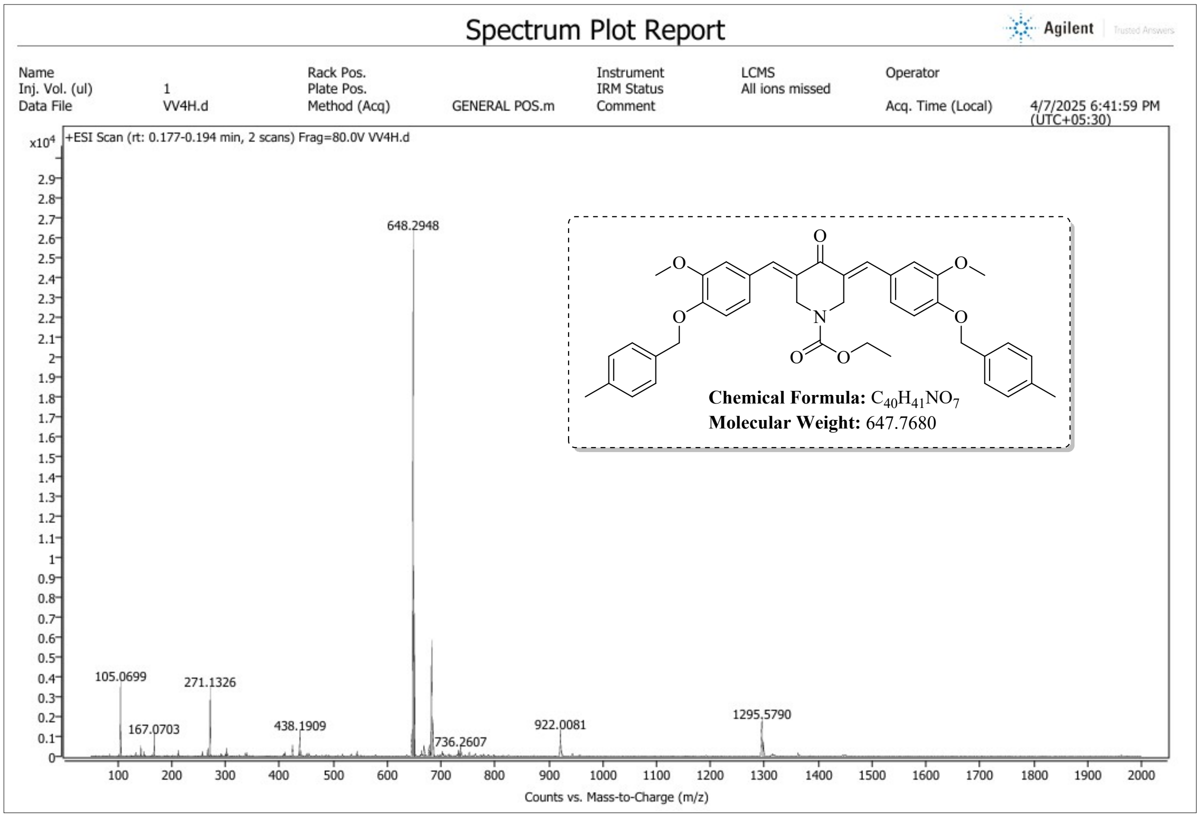

Figure S27: HR-MS Spectrum of compound **4h**

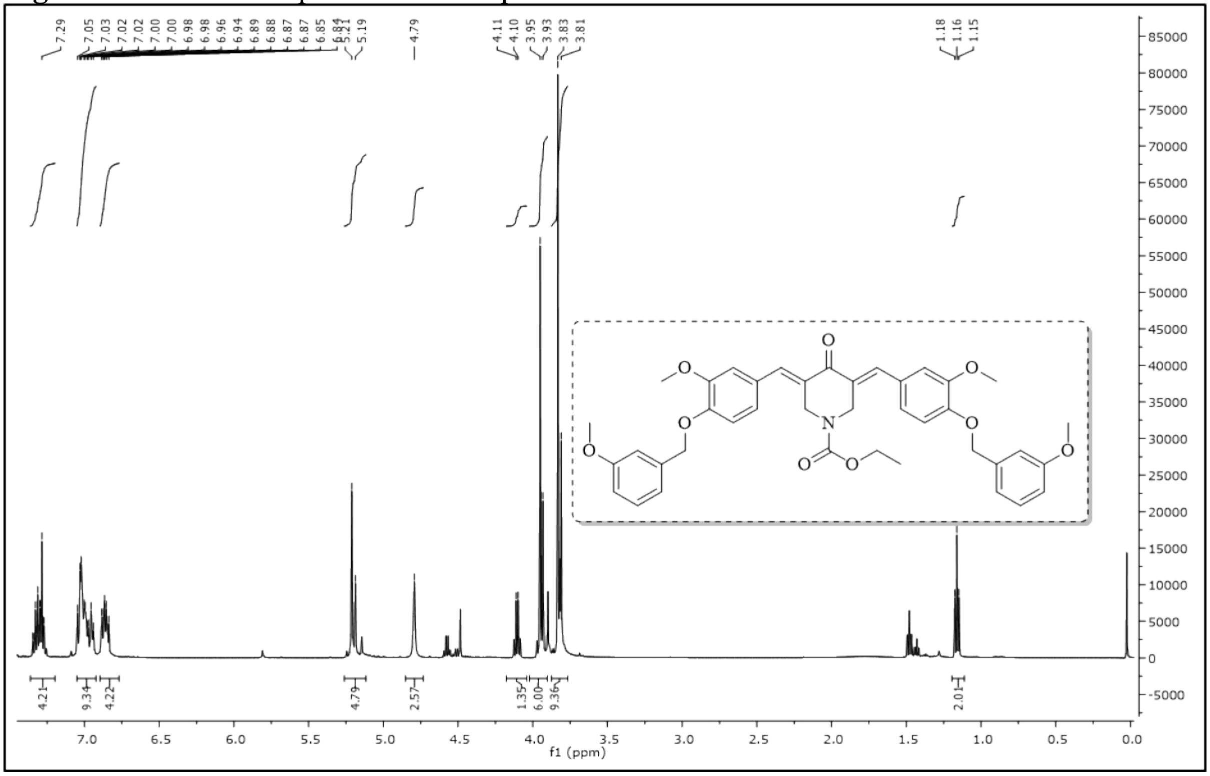

Figure S28: ^{1}H NMR Spectrum of compound **4i** [500 MHz, Solvent-$CDCl_3$]

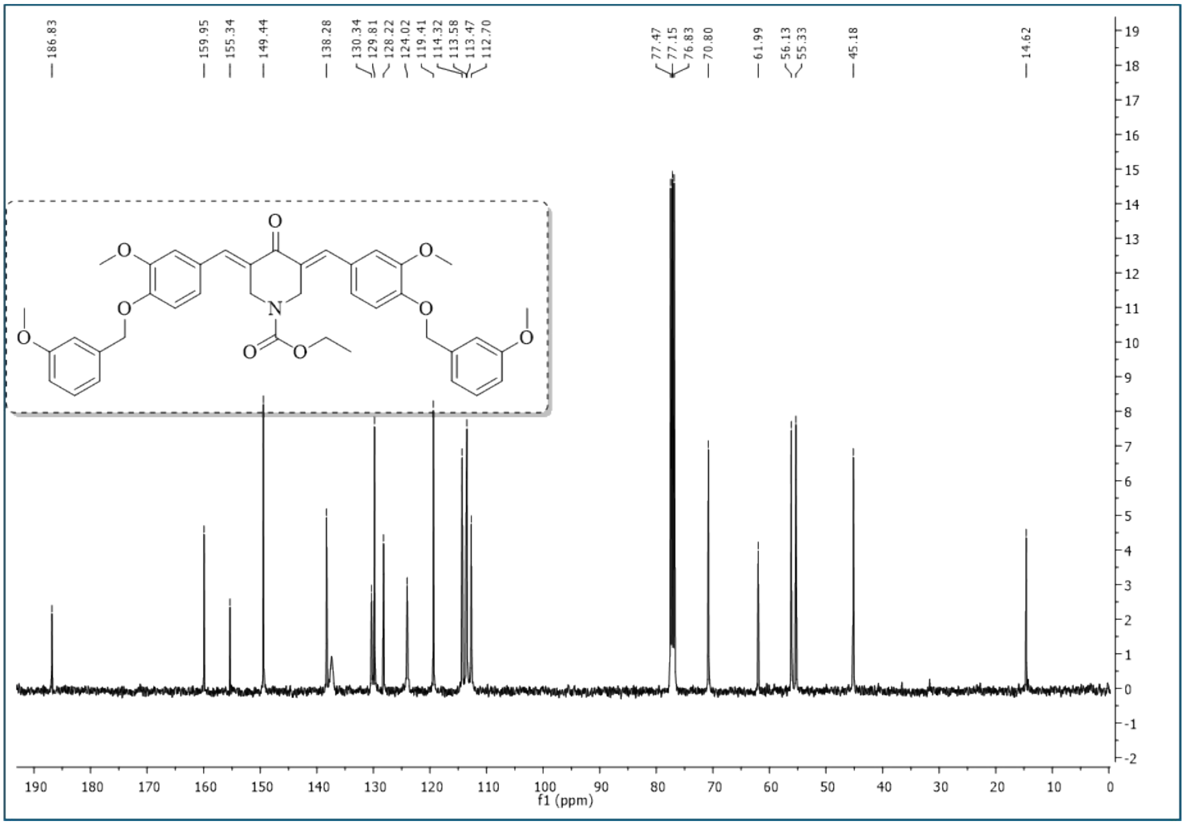

Figure S29: ^{13}C NMR Spectrum of compound **4i**.[125 MHz, Solvent-$CDCl_3$]

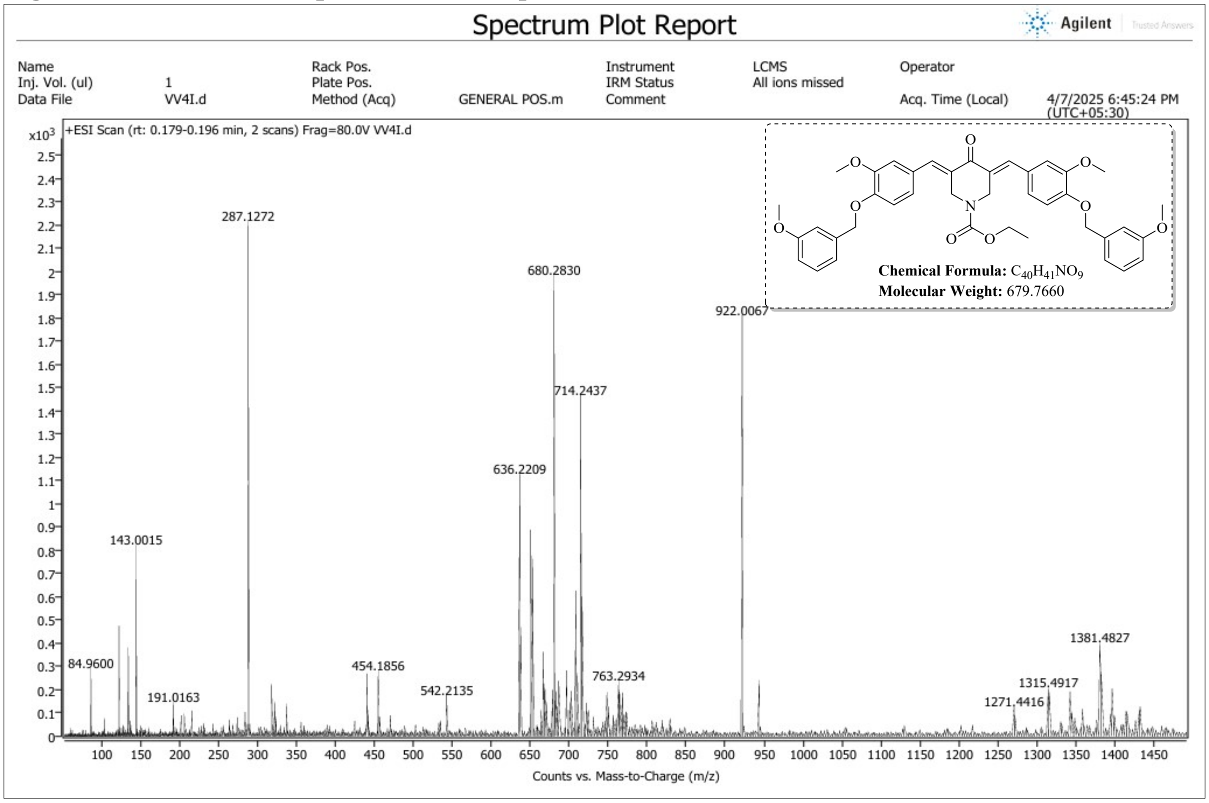

Figure S30: HR-MS Spectrum of compound **4i**

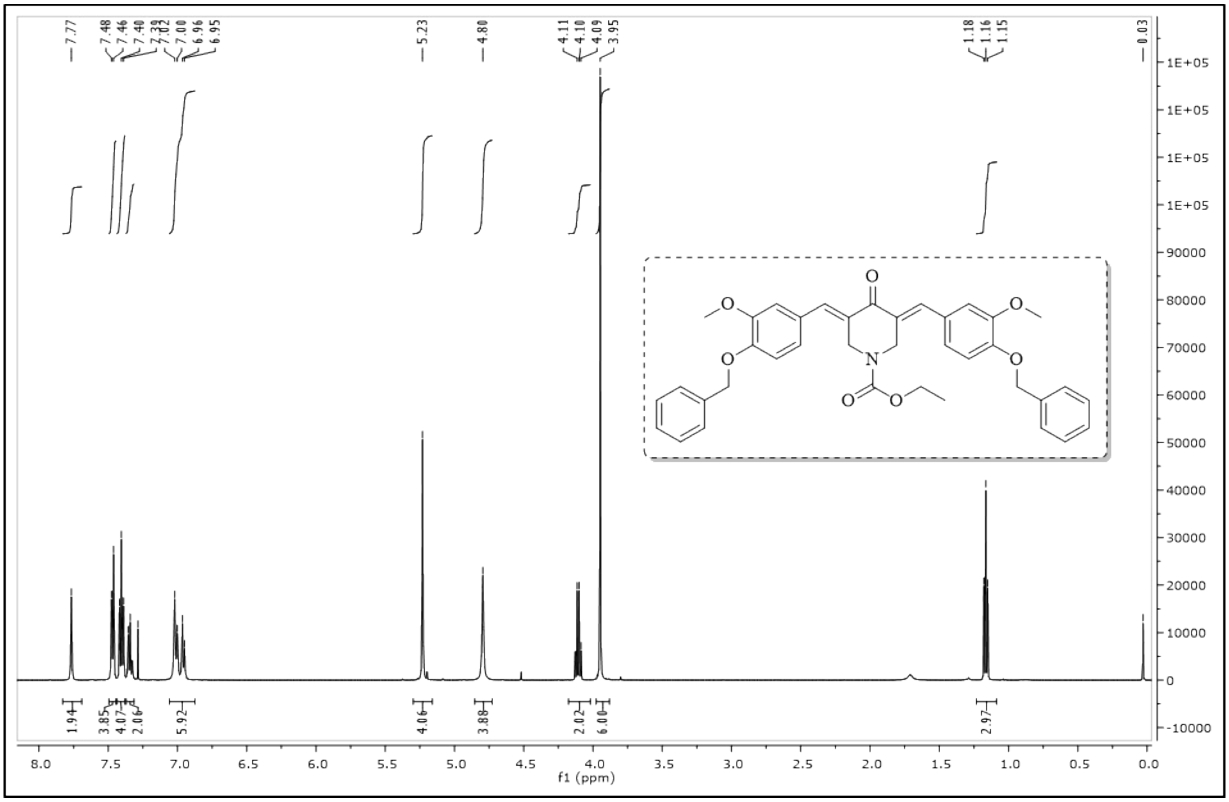

Figure S31: ^{1}H NMR Spectrum of compound **4j** [500 MHz, Solvent-$CDCl_3$]

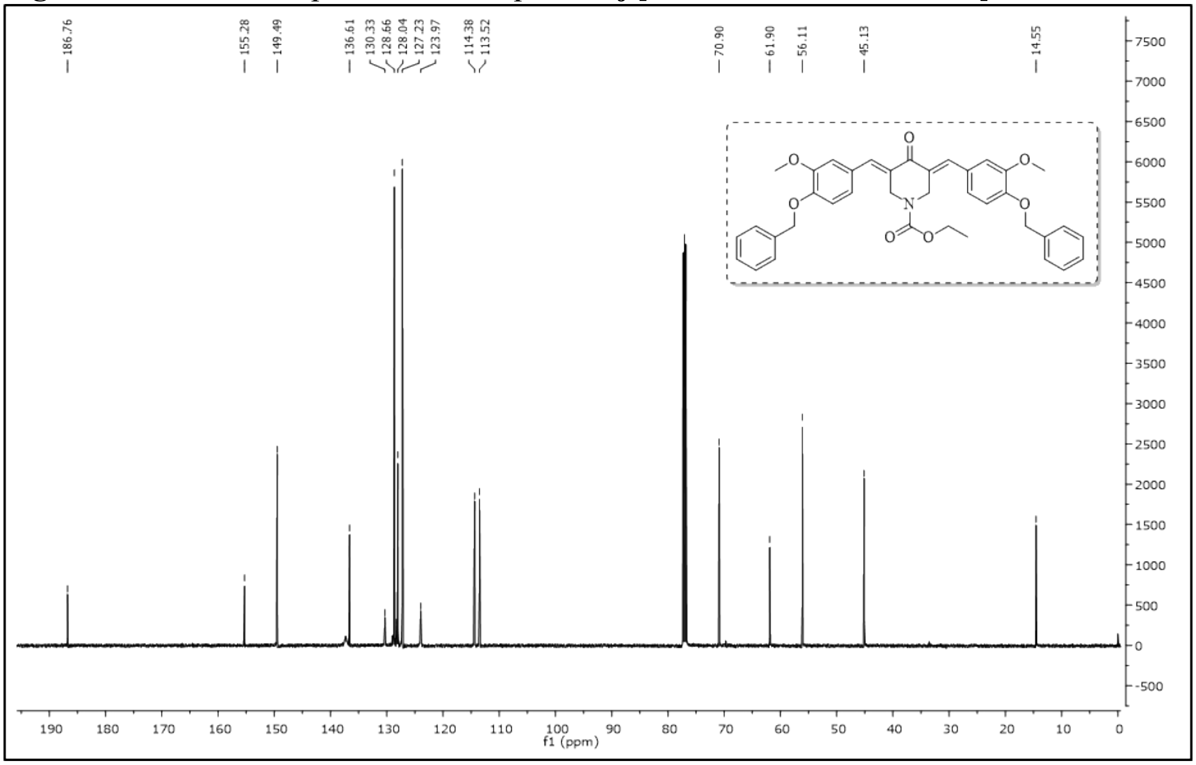

Figure S32: ^{13}C NMR Spectrum of compound **4j**. [125 MHz, Solvent-$CDCl_3$]

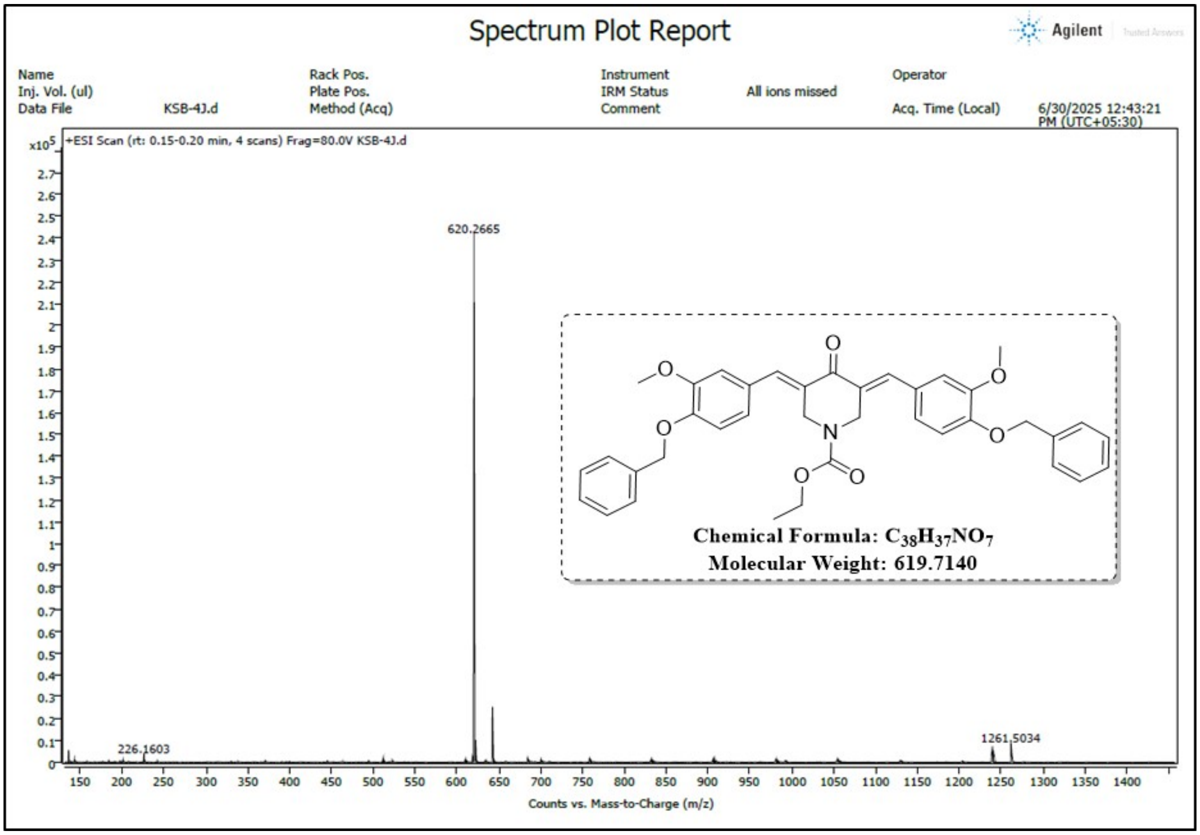

Figure S33: HR-MS Spectrum of compound **4j**

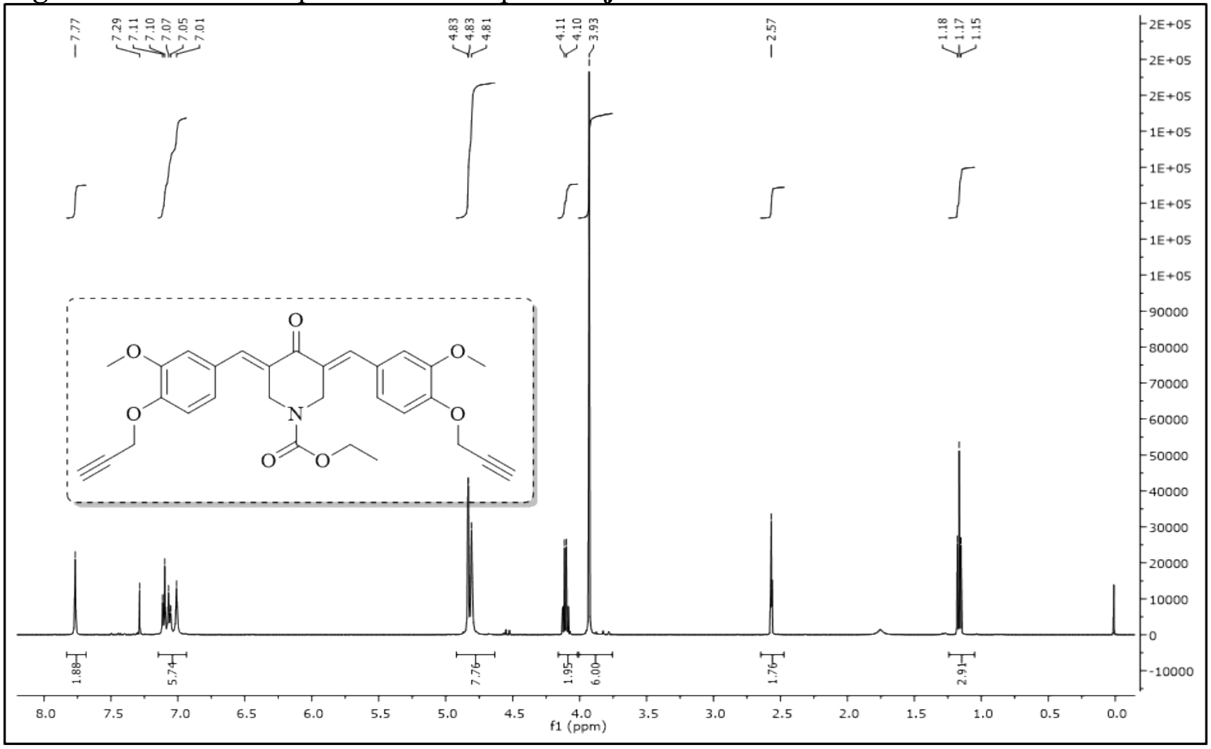

Figure S34: ^{1}H NMR Spectrum of compound **4k** [500 MHz, Solvent-$CDCl_3$]

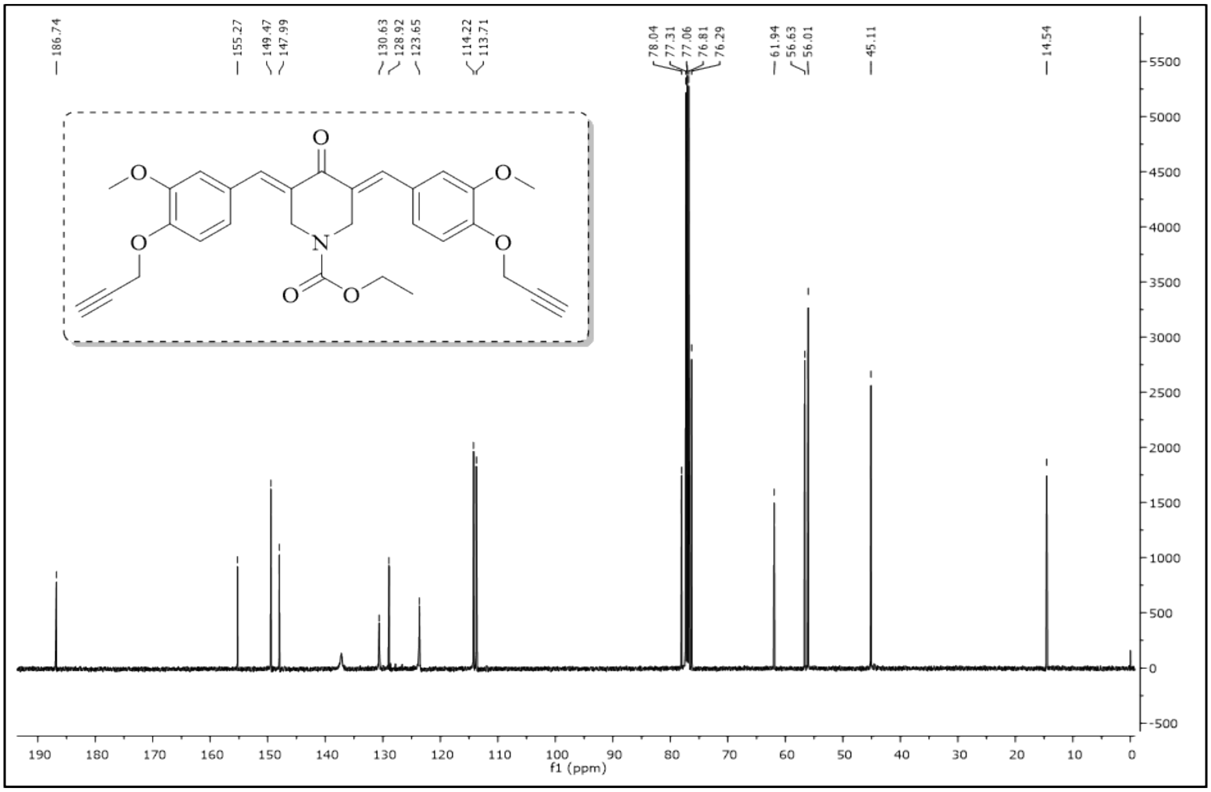

Figure S35: ^{13}C NMR Spectrum of compound **4k**. [125 MHz, Solvent-$CDCl_3$]

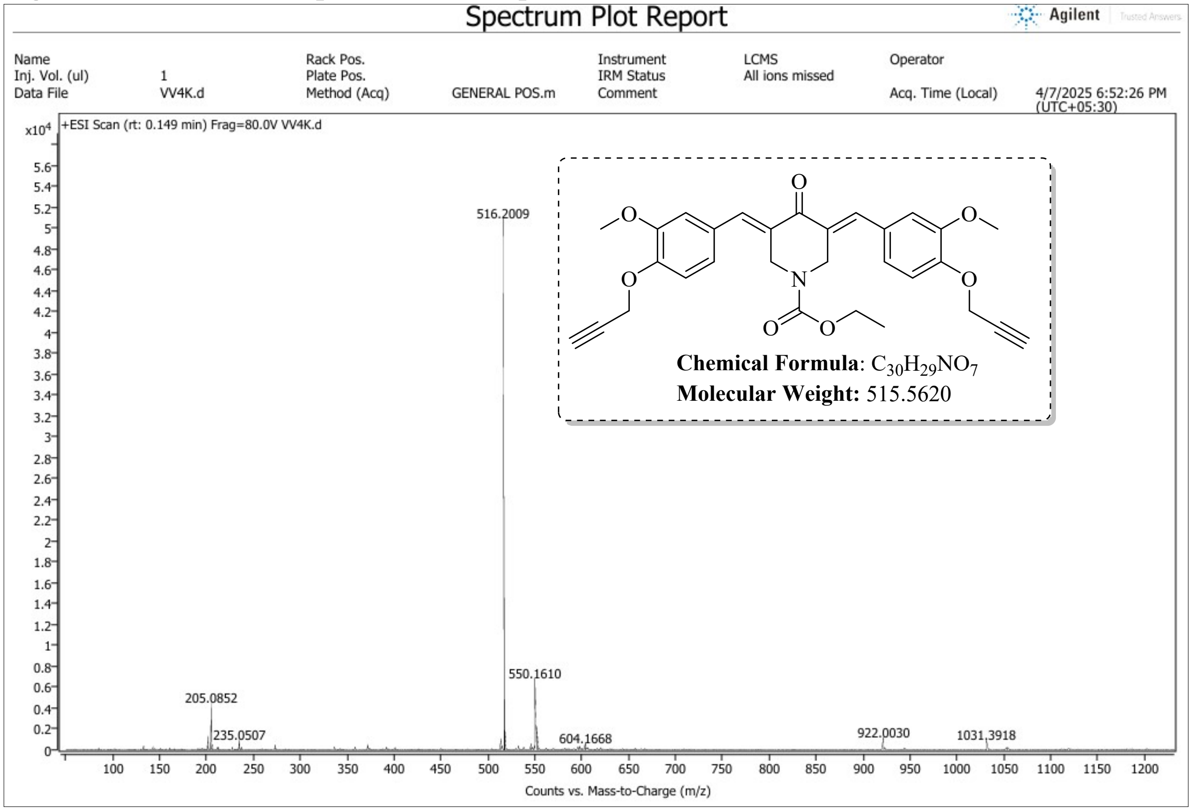

Figure S36: HR-MS Spectrum of compound **4k**

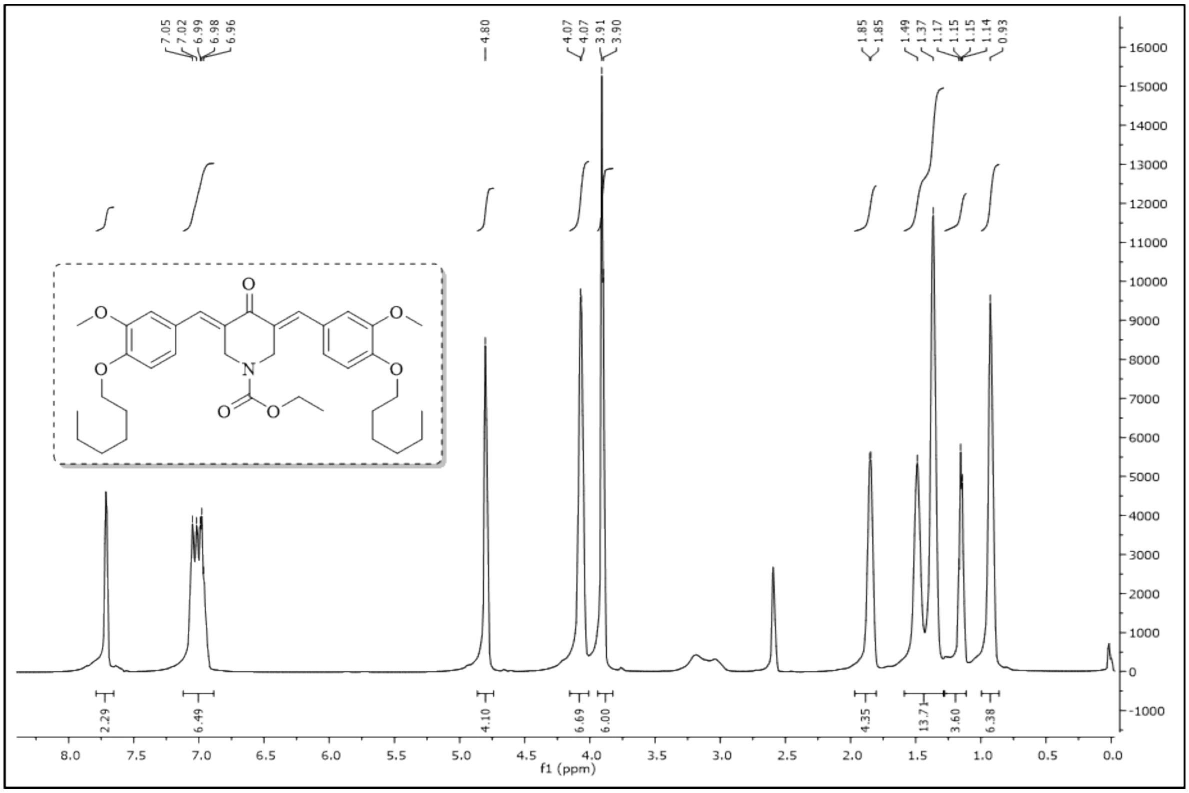

Figure S37: ^{1}H NMR Spectrum of compound **4l** [500 MHz, Solvent-$CDCl_3$]

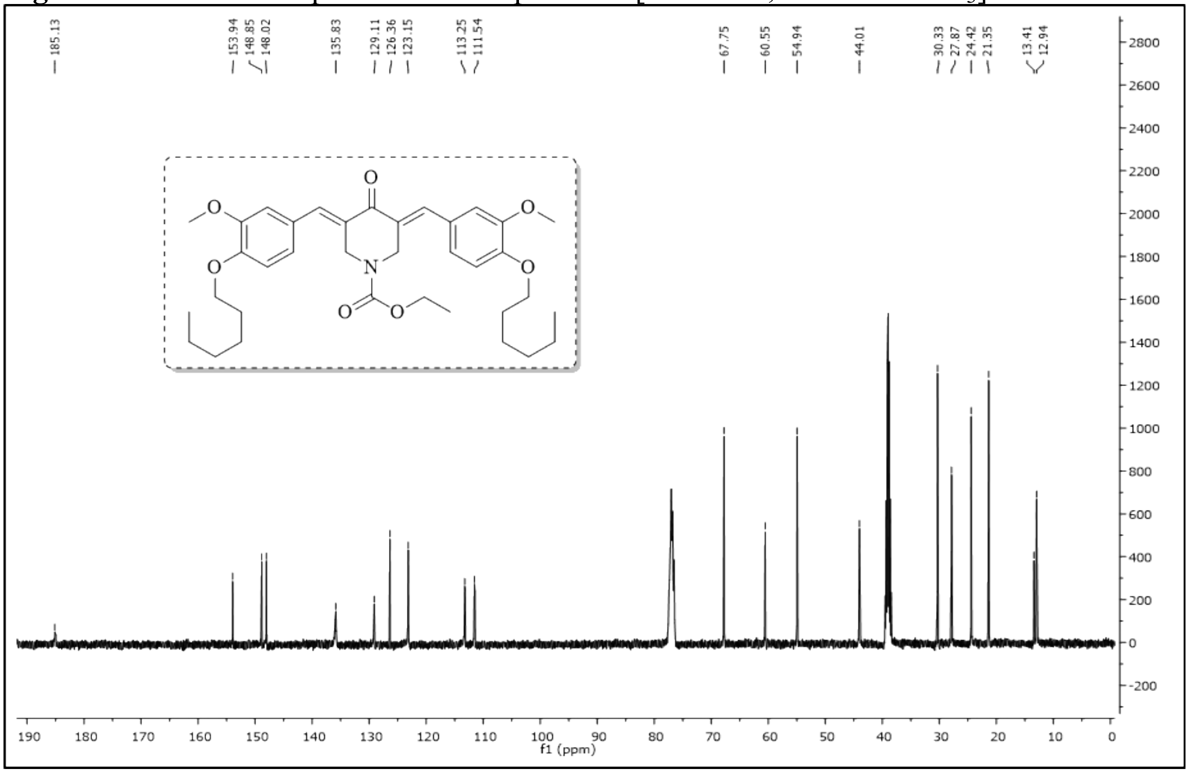

Figure S38: ^{13}C NMR Spectrum of compound **4l**. [125 MHz, Solvent-$CDCl_3$]

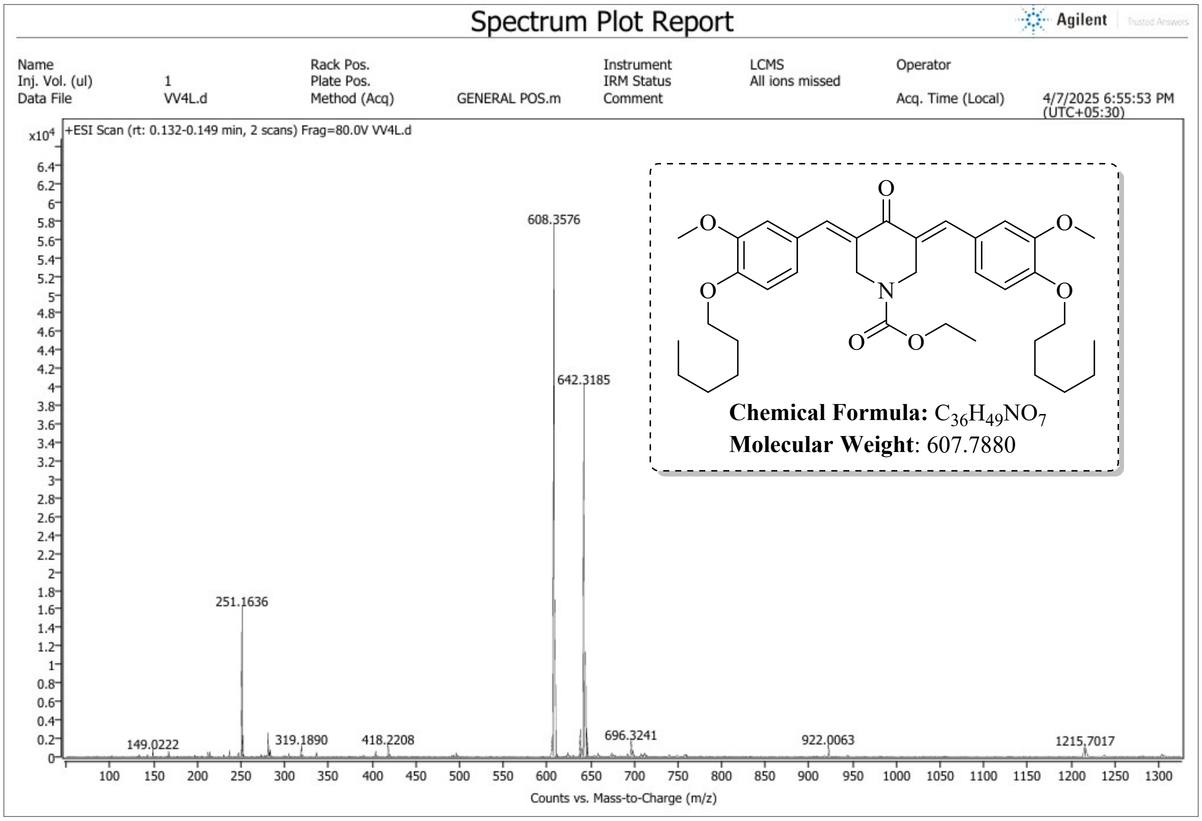

Figure S39: HR-MS Spectrum of compound **4l**

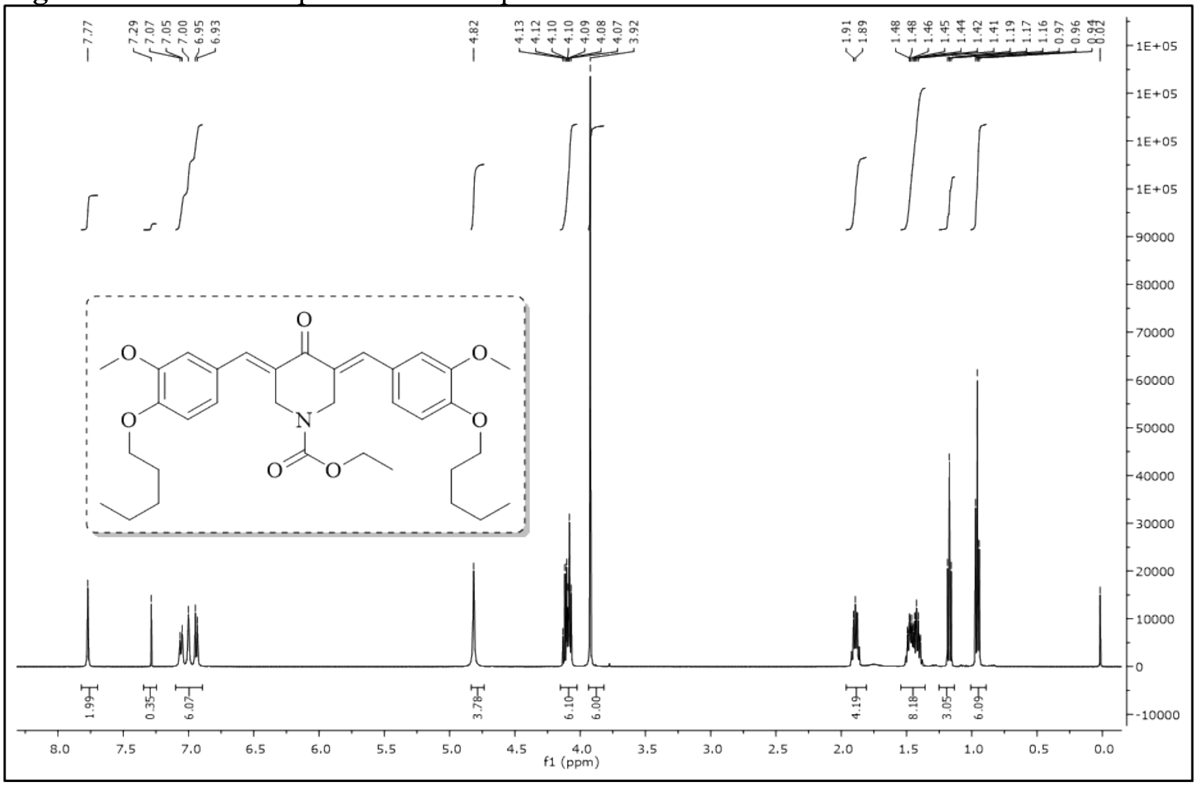

Figure S40: ^{1}H NMR Spectrum of compound **4m** [500 MHz, Solvent-$CDCl_3$]

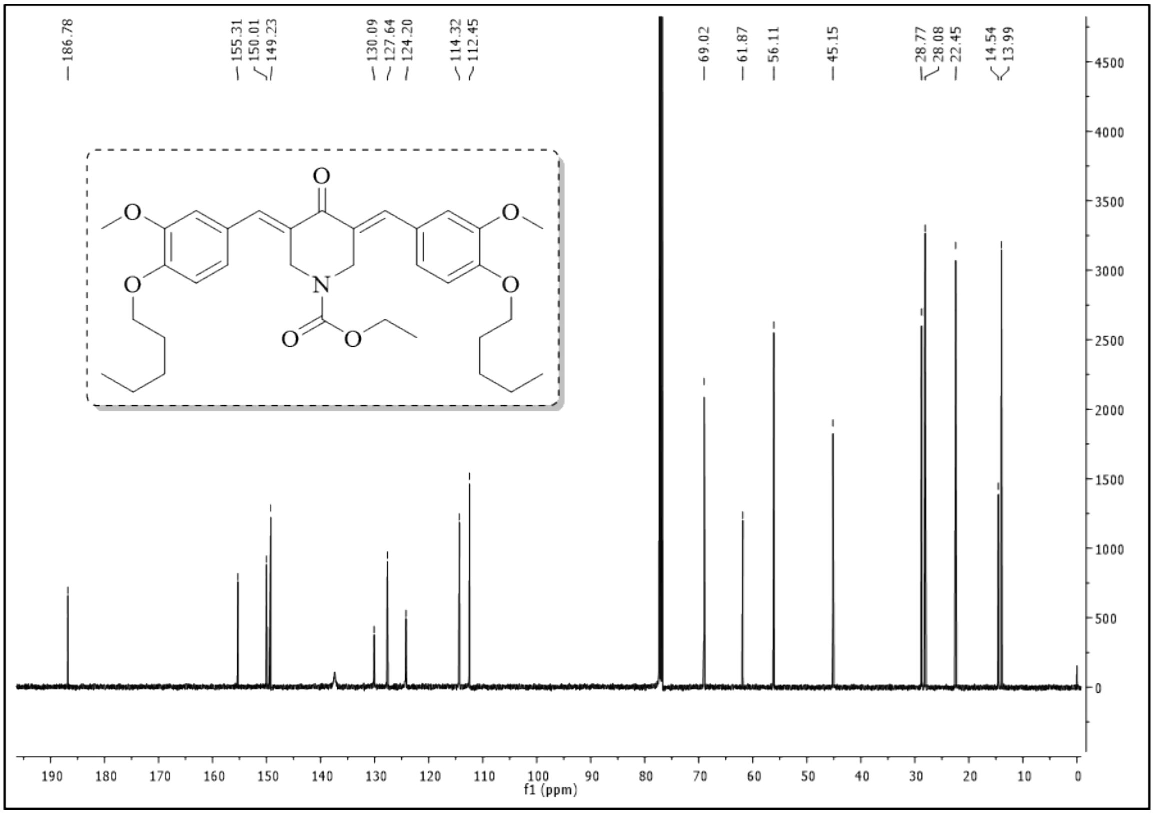

Figure S41: ^{13}C NMR Spectrum of compound **4m**. [125 MHz, Solvent-$CDCl_3$]

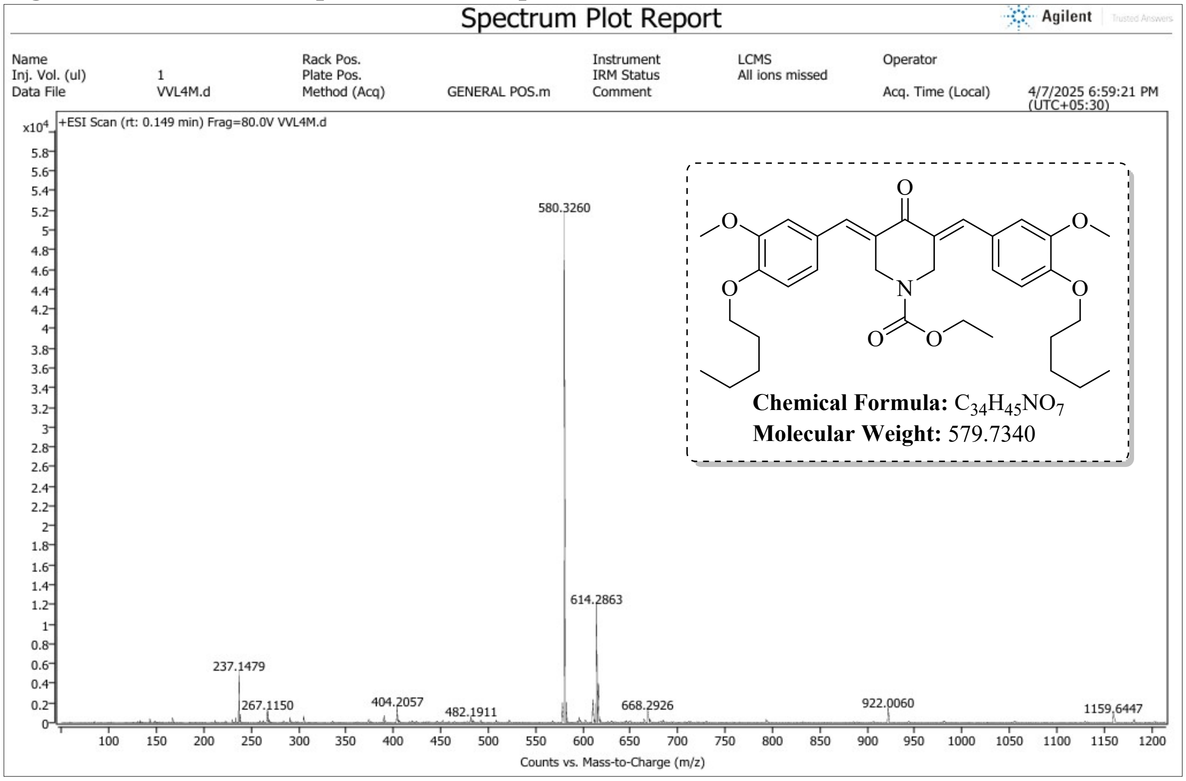

Figure S42: HR-MS Spectrum of compound **4m**

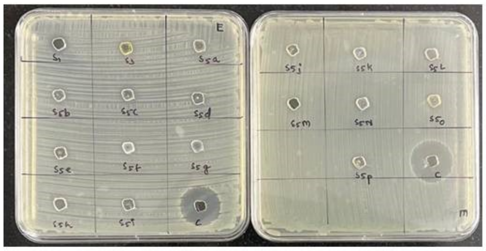

Escherichia coli (MTCC 730)

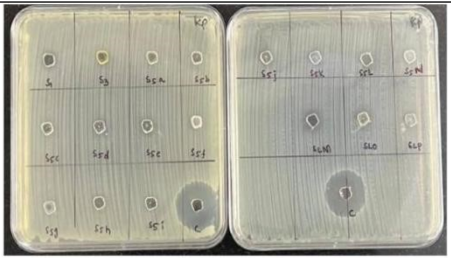

Klebsiella pneumonia (MTCC109)

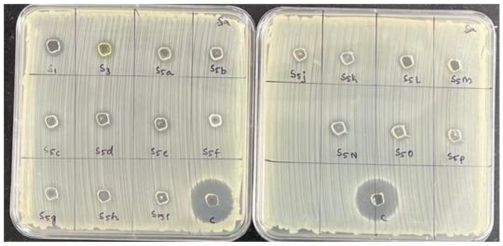

Staphylococcus aureus (MTCC 96)

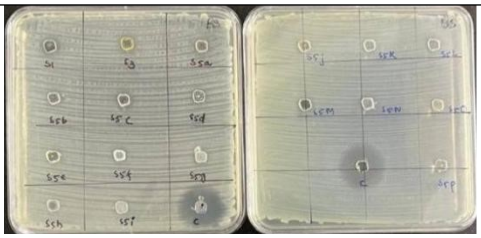

Bacillus subtilis (MTCC 121)

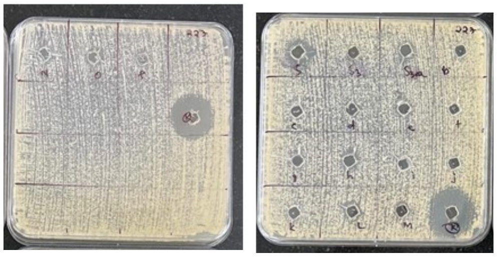

Candida albicans (MTCC-227)

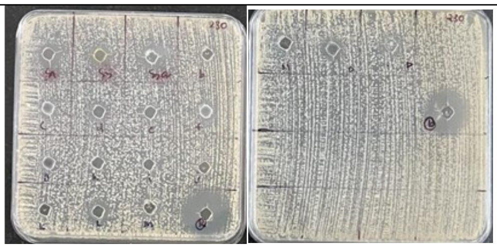

Candida tropicalis (MTCC 230)

Figure S43. Representative images of samples with standards **-**24 hrs (antibacterial and antifungal activity) using zone of inhibition study at 1 mg/mL

Table S1. In vitro cytotoxicity activity of 4K on Chinese Hamster Ovary cells (CHO-K1) in vitro

Compound Name	(IC_{50} in µM)
	**CHO-K1**
**4k**	13.5±1.9
**Mitomycin-C (Standard)**	13.1 ± 0.68

Cell lines CHO K1- Chinese hamster ovary cell line
